# Direct 3D printed biocompatible microfluidics: assessment of human mesenchymal stem cell differentiation and cytotoxic drug screening in a dynamic culture system

**DOI:** 10.1186/s12951-022-01737-7

**Published:** 2022-12-27

**Authors:** Oliver Riester, Stefan Laufer, Hans-Peter Deigner

**Affiliations:** 1 Institute of Precision Medicine, Furtwangen University, Jakob-Kienzle-Strasse 17, 78054 Villingen-Schwenningen, Germany; 2 Institute of Pharmaceutical Sciences, Department of Pharmacy and Biochemistry, Eberhard-Karls-University Tuebingen, Auf Der Morgenstelle 8, 72076 Tübingen, Germany; 3 Tuebingen Center for Academic Drug Discovery & Development (TüCAD2), 72076 Tübingen, Germany; 4 Faculty of Science, Eberhard-Karls-University Tuebingen, Auf Der Morgenstelle 8, 72076 Tübingen, Germany; 5 EXIM Department, Fraunhofer Institute IZI (Leipzig), Schillingallee 68, 18057 Rostock, Germany

**Keywords:** Microfluidic, Direct 3D printed, Drug screening system, Stem cell differentiation, Microfluidic long-term cell culture, Ready-to-use, Biocompatible

## Abstract

**Background:**

In vivo-mimicking conditions are critical in in vitro cell analysis to obtain clinically relevant results. The required conditions, comparable to those prevalent in nature, can be provided by microfluidic dynamic cell cultures. Microfluidics can be used to fabricate and test the functionality and biocompatibility of newly developed nanosystems or to apply micro- and nanoelectromechanical systems embedded in a microfluidic system. However, the use of microfluidic systems is often hampered by their accessibility, acquisition cost, or customization, especially for scientists whose primary research focus is not microfluidics.

**Results:**

Here we present a method for 3D printing that can be applied without special prior knowledge and sophisticated equipment to produce various ready-to-use microfluidic components with a size of 100 µm. Compared to other available methods, 3D printing using fused deposition modeling (FDM) offers several advantages, such as time-reduction and avoidance of sophisticated equipment (e.g., photolithography), as well as excellent biocompatibility and avoidance of toxic, leaching chemicals or post-processing (e.g., stereolithography). We further demonstrate the ease of use of the method for two relevant applications: a cytotoxicity screening system and an osteoblastic differentiation assay. To our knowledge, this is the first time an application including treatment, long-term cell culture and analysis on one chip has been demonstrated in a directly 3D-printed microfluidic chip.

**Conclusion:**

The direct 3D printing method is tested and validated for various microfluidic components that can be combined on a chip depending on the specific requirements of the experiment. The ease of use and production opens up the potential of microfluidics to a wide range of users, especially in biomedical research. Our demonstration of its use as a cytotoxicity screening system and as an assay for osteoblastic differentiation shows the methods potential in the development of novel biomedical applications. With the presented method, we aim to disseminate microfluidics as a standard method in biomedical research, thus improving the reproducibility and transferability of results to clinical applications.

**Graphical Abstract:**

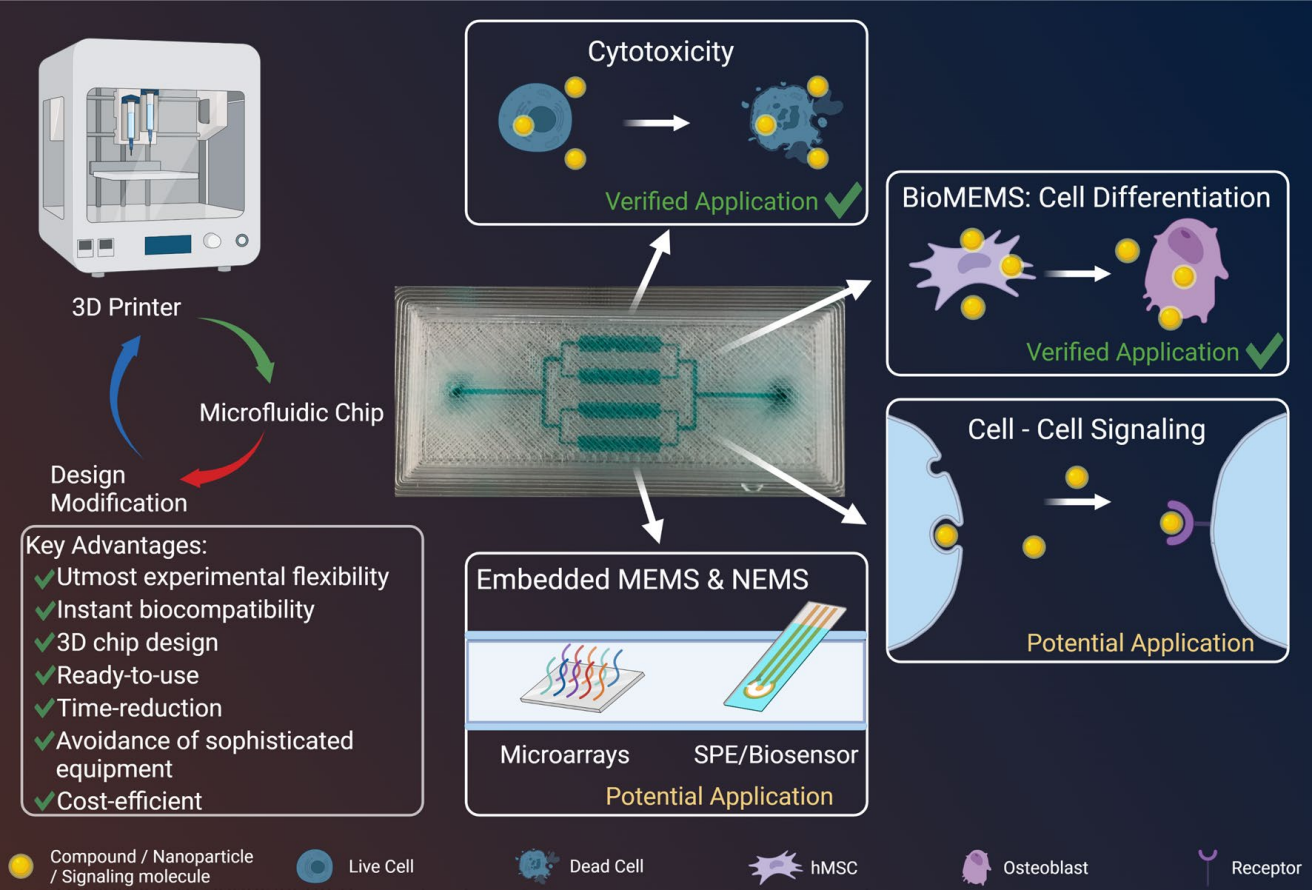

**Supplementary Information:**

The online version contains supplementary material available at 10.1186/s12951-022-01737-7.

## Background

**Fig. 1:**
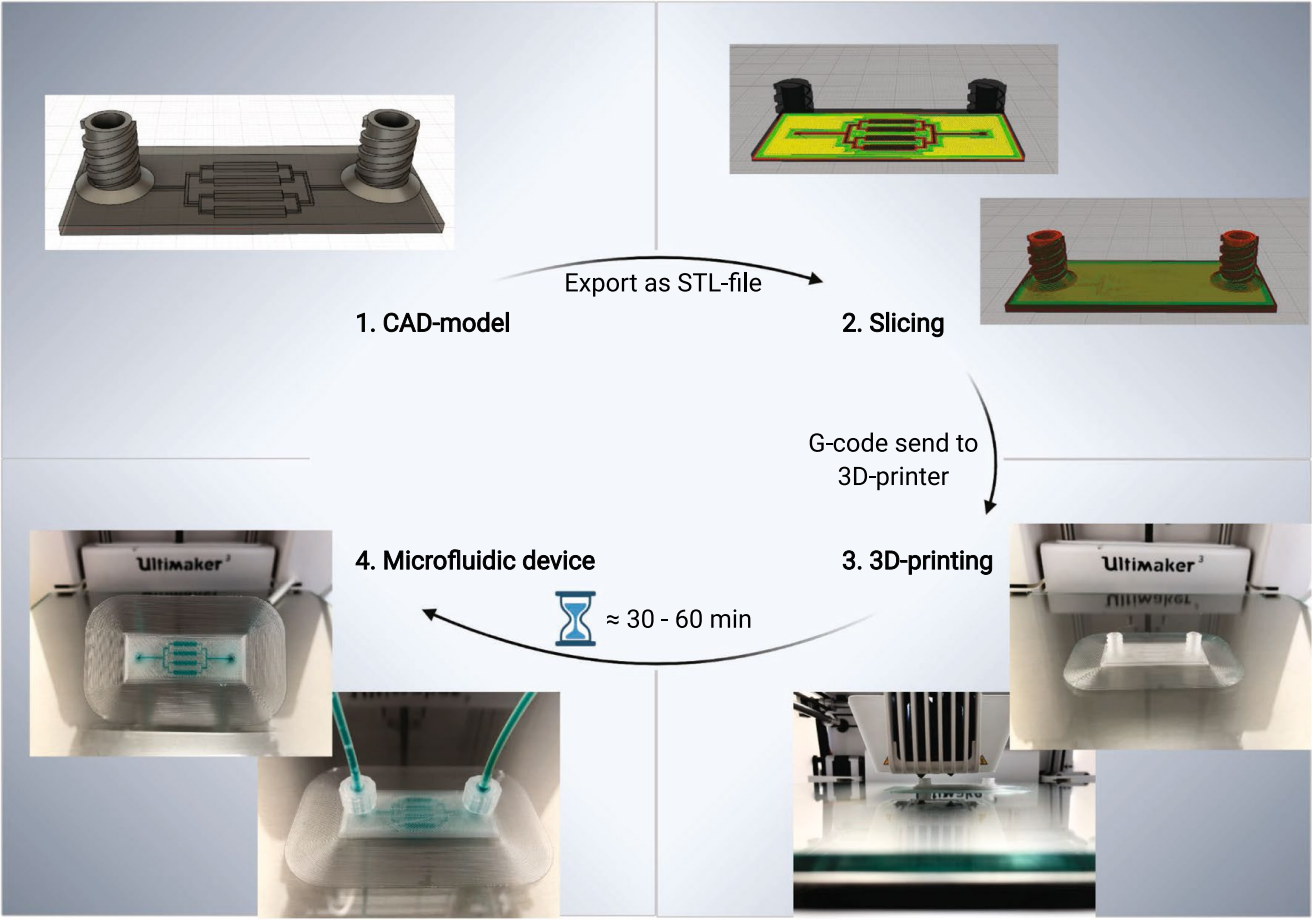
Workflow for direct 3D printing of a microfluidic chip. **1** CAD model: a CAD model of the device is created. This includes the intended channel geometry. **2** Slicing: the CAD model is exported as an STL file and loaded in the slicer software Ultimaker Cura. The software calculates the printing movements for each layer on the basis of the entered parameters and saves it in a G-code file. **3** 3D printing: the G-code is sent to the 3D printer, which prints the device according to the parameters listed in the file. **4** Microfluidic device: the printing process is complete after approximately 30–60 min, depending on the device size. The printed microfluidic device is ready to use. Figure created with BioRender.com

The ability of stem cells to differentiate into specialized cell types is considered one of the most promising ways to replace damaged tissue or even entire organs, and to provide effective treatments for numerous diseases [1, 2]. However, despite major research efforts in this field over the past decades, the application of stem cell therapies beyond clinical trials still faces several practical challenges, including stem cell origin, isolation, expansion, stability and efficient protocols for targeted differentiation [3, 4]. The systems investigated are becoming increasingly complex, particularly in the fields of system- and cell biology, wherein many systems interact and are often not fully understood [5–8]. These problems led to the development of high-throughput methods as well as the software necessary to create, treat and analyze the large numbers of test samples [9–11]. Stem cell differentiation is one of these complex processes influenced by a variety of extrinsic and intrinsic interactions [12–14]. In targeted stem cell differentiation, not only single molecules or mechanisms, but a multitude of extrinsic and intrinsic interacting systems are crucial in ultimately determining the lineage of differentiation. Microfluidic systems help in targeted differentiation, analysis and cultivation of stem cells by creating realistic microenvironments or by improving the predictability of biological assays. According to Ertl et al., microfluidic devices offer many advantages “to overcome most of the challenges associated with stem cell identification, expansion and differentiation, with the greatest advantage being that lab-on-a-chip technology allows for the precise regulation of culturing conditions, while simultaneously monitoring relevant parameters using embedded sensory systems” [15]. Microfluidic applications range from the production of nanomaterials and chemical reactions to biosensors, diagnostic systems and high-throughput screening systems [16–22]. The characteristics of a microfluidic system with laminar flow and short diffusion paths optimize these applications in many aspects, for example by enabling users to manipulate liquids in a targeted manner and achieve near-ideal mixing ratios without additional energy input. Microfluidic devices have shown promising results in life sciences, such as decreased consumption of expensive and limited materials, or the establishment of manipulable dynamic culture systems superior to static cell culture systems [23–26]. However, one factor in particular has made these systems difficult to obtain for many researchers and therefore rarely used: experiments or applications often require customized devices, thus hindering standardized commercial manufacturing [27]. Consequently, many applications are limited by the standardized chips available on the market, which are often simply designed to be suitable for as many applications as possible. For more demanding applications, users must either have equipment for in-house production or rely on custom-made chips [28, 29]. Both options are often costly, thus discouraging many users. On-site production is preferable solely because it eliminates delivery times, thus allowing for immediate adjustments and improvements, particularly in prototype production. The current standard procedures for the creation of individualized microfluidic devices are indirect casting processes, photolithography or e-beam lithography; these labor- and equipment-intensive methods require extensive manual work [30, 31]. Soft lithography is another method for producing micro- and nanostructures that is more cost-effective than photolithography, because it no longer requires a clean room. However, the costs still prevent widespread use, and the method remains labor intensive [32, 33]. Another disadvantage of these techniques is that they can only be used to produce 2D chip designs, and even here they require experienced personnel, as several layers have to be joined manually. Nevertheless, these methods remain in use because they have achieved the best accuracy to date and have produced structures on the scale of several nanometers [30]. In contrast, micropaper-based analytical devices (µPADs) are very well suited when high accuracy at nanometer and micrometer scale is not required. The idea underlying µPADs is to make microfluidic systems ready for mass production of simple and rapid diagnostic tests. The low accuracy and the generation of only 2D chips is sufficient for many applications but limits the design of more advanced chips [34]. Given the aforementioned methods, the production of complex 3D microfluidic chips is not a trivial challenge. 3D printing emerged as an alternative method for the production of microfluidics. Commercially available 3D printers currently have sufficient accuracy to print channel widths of several hundred micrometers, as a result of intensive development in recent decades [35, 36]. Several 3D printing technologies are available, such as inkjet printing [37], stereolithography (SLA) [38], digital light processing [31] and fused deposition modeling (FDM) [39]. FDM is the most widely used 3D printing technology, owing to its simplicity: only the polymer filament is needed as a resource in the process, in contrast to other printing technologies in which the polymers are dissolved in a solution or are present as a resin and polymerize in the process [35, 40]. FDM 3D printers are popular not only because of their ease of use but also because they do not require additional substances such as photoinducers, which are often toxic and leak from devices over time [41]. In addition, a wide range of polymers can be purchased, thereby avoiding limitations in material selection. For the production of microfluidic chips by 3D printing, two production options are available: indirect production [42], in which a negative form is printed for a casting process, and direct production, in which the computer-aided design (CAD) model is converted directly to the microfluidic chip. Indirect production, as shown by He et al. [43], results in highly transparent and biocompatible chips suitable for cell culture and analytical assays. However, they are limited by two factors: first, the minimum component size is determined by the width of the printable line. Second, the printed negative mold must be stable and elastic enough not to be deformed during casting. This is particularly challenging for large and complex 3D structures connected by small channels. In contrast, directly printed chips do not have any stability problems of a negative mold, but have lower material transparency depending on the manufacturing process, which hampers optical measurements and observations. Bressan et al. [44] created a mixture of both fabrication methods by inserting a prefabricated transparent window made of poly (methyl methacrylate) (PMMA) into a chip printed from poly (lactic acid) (PLA). Thus, the problem of transparency was solved, but replaced by a vulnerability at the interface of the two materials, leading to leakage. A different approach to achieve the necessary transparency is to optimize the printing parameters, as shown for example by Tothill et al. for PLA [45]. However, these parameters are material-specific and must therefore be investigated once for the respective polymer before application. Most previous studies on direct FDM 3D printing either show simple applications with only one component on a chip and channel sizes in the millifluidic range, or focus on the achievable accuracy without showing suitability for biological applications [37, 46]. In this study, we therefore demonstrate the fabrication of biocompatible microfluidic chips with structures of 100 µm and smaller using three relevant polymers and that experiments from preparation to cell culture and analysis can be performed on a single chip by combining multiple components. We studied the polymers—PLA, PMMA and polycarbonate (PC)—which are frequently used in the field of microfluidics and cover a wide range of applications with their advantages and characteristics, as listed in Table 1. PLA, for example, is particularly suitable for prototype construction or the generation of vascular scaffolds [47], owing to its simple handling, good availability and high accuracy. PMMA, in contrast, has excellent biocompatibility and modifiability with different chemical groups [48–50]. The third polymer, PC, has high mechanical stability, as well as temperature resistance and chemical stability against acids [51], and is ideal for applications with high temperatures up to 140 °C [52]. Here, we demonstrated the generation of microfluidic chips by using the mentioned polymers, without a need for additional support materials. In the device generation, we used the direct 3D printing principle, as shown in Fig. 1, which allowed us to generate ready-to-use microfluidic devices from the CAD model with just several clicks. In this context, we demonstrate the fabrication of 3D chip designs and widely used microfluidic structures, as well as their application in microfluidic cytotoxicity and stem cell differentiation assays.

**Table 1:** Characteristics and possible applications of poly (lactic acid) (PLA), poly (methyl methacrylate) (PMMA) andpolycarbonate (PC) in the generation of microfluidic devices

Polymer	Characteristics	Possible applications
PLA	Advantage: • Easy to use • Recyclable • Transparent • Low auto-fluorescence [53] • No absorption of small molecules [53] Disadvantage: • Hydroscopic material – swelling in water • Lactic acid as degradation product • Can show cytotoxic effects	Prototype design Organ on-chip [53] Cell culture [53] Incorporation of Microelectrodes [54]
PMMA	Advantage: • Transparent • Biocompatible [48, 49] • Surface modification [50] • Heat resistant up to 90 °C [55] • Impermeable to air [56] • UV-resistant • Resistant to many acids, bases, alcohols, oils and fats [57] Disadvantage: • Not resistant to many organic solvents	PCR-on-chip [50] Lab-on-chip [58–60] DNA/Protein analysis [61, 62] Electrochemical detection [54, 63] Colorimetric assays Assembling of micro and nanoparticles [44, 64]
PC	Advantage: • Transparent • Heat resistant up to 140 °C [52] • Acid resistance [51] • Naturally hydrophilic surface [65] • Surface modification [66] Disadvantage: • Sensitive during printing process: environmental conditions • Poor adhesion properties during the printing process	Electrochemical detection [54, 67] Lab-on-chip [60, 67] PCR-on-chip [65, 68] Biomedical studies [68] Droplet generation [65]

## Results

### Resolution of direct 3D printed microfluidic channels

**Fig. 2:**
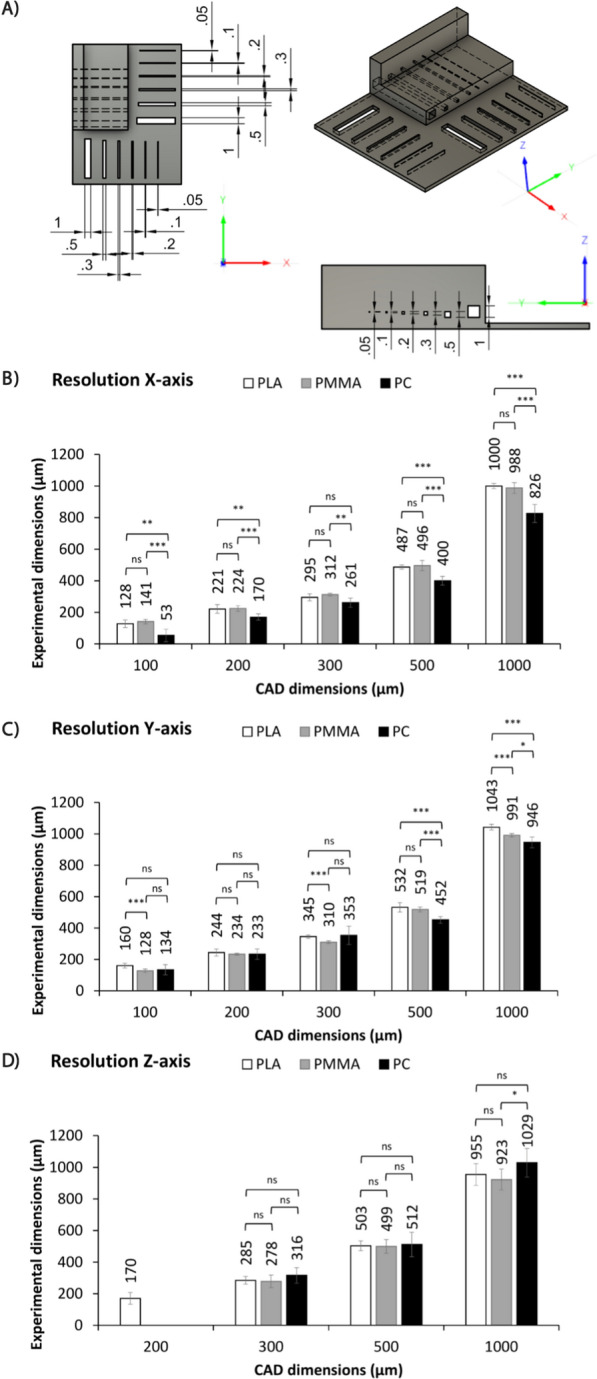
Measurement of widths and heights of FDM printed micro channels. The printed test objects were compared with the CAD model to evaluate the achievable resolutions for poly (lactic acid) (PLA), poly (methyl methacrylate) (PMMA) and polycarbonate (PC). **A** CAD model of the test device. Dimensions are in mm. **B** Resolution of channel widths along the X-axis of the 3D printer. Dimensions are in µm. **C** Resolutions of channel width along the Y-axis of the 3D printer. Dimensions are in µm. **D** Resolution of channel height along the Z-axis of the 3D printer. Dimensions are in µm. Values are shown as mean ± SD of 3 devices, 3 measurements per device. Statistical significance was analyzed with Two-way ANOVA and Tukey post-hoc test (n s, not significant; *p < 0.05; **p < 0.01; ***p < 0.001)

The direct FDM 3D printing of microfluidic devices is influenced by various parameters including the accuracy of the printer step motors, the printer head nozzle diameter, the environmental temperature and even the humidity. In this study, four parameters were optimized for each polymer: printing temperature (ϑ), printing speed (v), layer height (h) and fan speed (fan). Each parameter is directly involved in the printing process and has a significant influence on the obtained dimensions of printed structures [37, 69, 70]. It was observed that ϑ and v had the strongest effect on the printing results (Additional file 1: Fig. S1–S15), while layer height and fan speed had a lesser influence. The former (h) showed an effect, particularly at low Z-resolution, because the structure must be sliced as an integer multiple of the layer height. Layer heights of 200 µm resulted in the loss of structures below 200 µm in the Z-direction or were sliced as if they were 200 µm structures. Very low layer heights, such as 25 µm, resulted in good slicing, but the printed layers were not uniform because the print head smudged the newly applied material. Layers of 100 µm provided a good compromise between both effects and were therefore used in further printing tests. The optimized parameters for the three tested polymers PLA, PMMA and PC are listed in Table 2. By applying the listed parameters, channel widths of 100 µm and channel heights of 300 µm were reproducibly generated, as shown in Fig. 2. The low standard deviations (Fig. 2) obtained across three different devices indicate good and consistent device to device performance. The polymers PLA and PMMA showed the best correlations between the CAD model and the obtained channel widths (Additional file 1: Fig. S16–S18), with only occasional significant differences between them. PC, on the other hand, mostly resulted in significantly smaller channels than specified, especially for channel widths of 500 µm (X: 400 ± 28 µm; Y: 452 ± 22 µm) and 1000 µm (X: 826 ± 22 µm; Y: 946 ± 35 µm). Printing channels with a width of 50 µm was also possible, but they occasionally merged and required post-processed manual verification of permeability (Additional file 1: Fig. S13). Therefore, they were excluded from the data shown.

**Table 2:** Optimized printing parameters for the generation of microfluidic devices with an Ultimaker 3 FDM 3D printer and Ultimaker Cura Slicer Software

Polymer	Printing temperature [°C]	Printing speed [mm s^−1^]	Layer height [mm]	Fan speed [%]
PLA	190	70	0.1	50
PMMA	245	70	0.1	50
PC	240	80	0.1	0

### 3D microfluidic structures

**Fig. 3:**
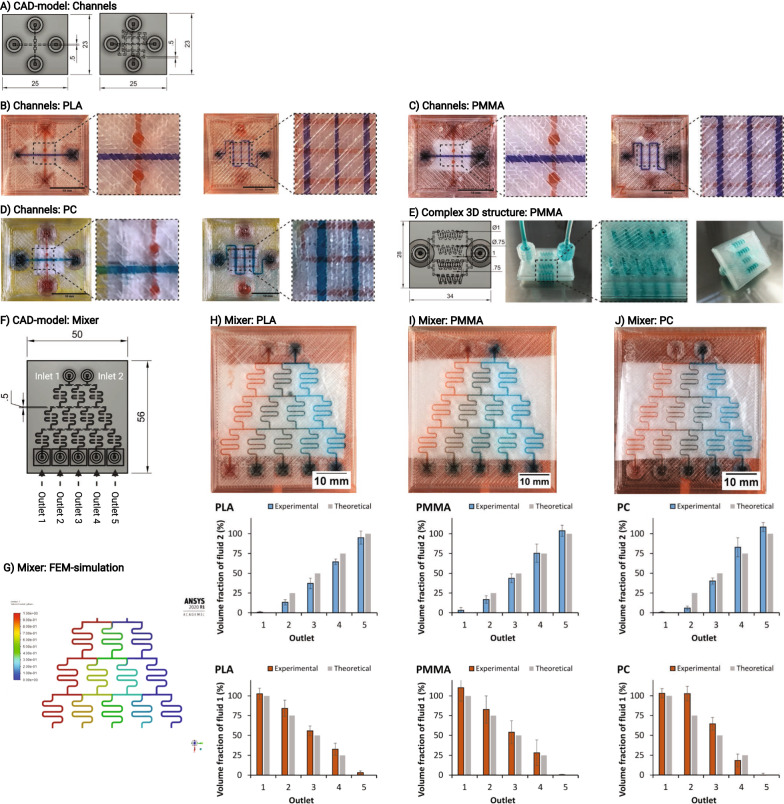
Possible designs of 3D printed microfluidic devices. **A** CAD models of channel test devices: the left shows two straight channels that cross each other with a bridge; the right shows two serpentine channels, one above the other. The distance between channels in the Z-direction is 0.2 mm. Dimensions are in mm. Test devices (Channels) 3D printed from **B** poly (lactic acid) (PLA), **C** poly (methyl methacrylate) (PMMA) and **D** polycarbonate (PC). **E** Complex 3D spiral structure printed from PMMA. **F** CAD model of a passive mixer design. Dimensions are in mm. **G** Mixing of two fluids in the designed mixing chip. Simulated with the finite element method (FEM) in Ansys 2020 R1 Academics with a flow of 0.2 ml/min. Fluid 1 is shown in red, fluid 2 in blue, and 1:1 mixing in green. Test devices (Mixer) 3D printed from **H** poly (lactic acid) (PLA), **I** poly (methyl methacrylate) (PMMA) and **J** polycarbonate (PC). Top: pictures of a microfluidic passive mixing devices with a flow of 0.2 ml/min. All Scale bars measure 10 mm. Middle: volume fraction of fluid 2 (blue) in outlets compared to theoretical value (gray), analyzed with A_640nm_ measurements. Bottom: volume fraction of fluid 1 (red) in outlets compared to theoretical value (gray), analyzed with A_490nm_ measurements. Values are shown as mean ± SD of 3 devices

It is beneficial to perform all steps of an experiment (preparation/treatment, cultivation, analysis) on one chip in order to achieve additional benefits for biomedical applications. Therefore, it is preferred to produce and combine several components and structures on one chip, resulting in customized chip designs. However, the production of customized devices is often time-consuming and expensive [71]. Thus, 3D printing enables new devices to be designed and adapted in a time-efficient, cost-efficient and customized manner. The practicality and advantages of 3D printed microfluidic systems was demonstrated by creating and testing three microfluidic chip designs. The generated chips are shown in Fig. 3. First, a chip with two intersecting serpentine channels with a spacing in the Z-direction of 0.2 mm was printed. Multiple layers of channels were successfully created on top of each other without leakage, separated by only two printed polymer layers in between. This design is advantageous because several channel structures can be stacked on top of each other, reducing the device´s size. The second chip generated consisted of two straight channels crossing each other with a bridge, demonstrating the feasibility of printing channels not only in the X- and Y-directions, but also in the Z-direction for all three polymers tested. This ability facilitates chip planning and the connection of channels, which need not be arranged next to each other as in 2D chip designs. The third chip design (Fig. 3E) shows a 3D spiral structure as an example of more complex structures, that are difficult to produce using traditional methods. This third chip design was printed with PLA, PMMA and PC (only PMMA shown), with PC causing channel closure and PLA and PMMA showing comparable results.

### Microfluidic concentration-gradient generator

A frequently used component in the preparation/treatment steps of microfluidic experiments are gradient generators. The passive mixer shown in Fig. 3F) was 3D printed from PLA, PMMA and PC with the parameters in Table 2. The absorbance of fluids eluted from test devices at outlets 1 to 5 was measured at 490 nm and 640 nm to calculate the fluid fraction of fluid 1 and fluid 2 for each device separately, as shown in Fig. 3H–J. The devices printed from PLA and PMMA showed good correlation between the theoretically expected volume fractions and the experimental volume fractions. With a maximum relative deviation of 9% compared with the theoretical value, PMMA showed better correlation than the PLA device, with a maximum relative deviation of 15%. As observed in the experiments for the achieved resolution, the devices printed from PC appeared to underperform, thus resulting in a maximum relative deviation of 21% with respect to the theoretically expected values. This could particularly observed in outlets 2 and 4, which show nearly the same volume fractions as outlets 1 and 5, respectively.

### Absorbance measurement on chip

**Fig. 4:**
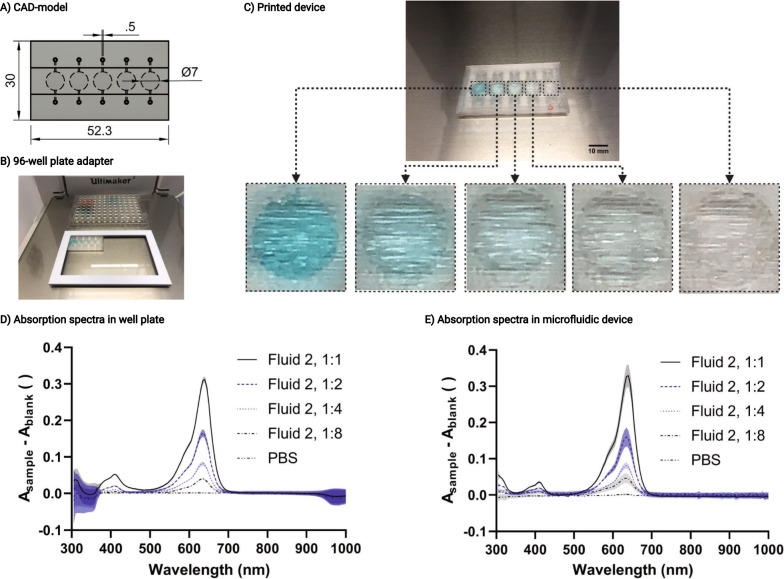
Absorbance measurement in a 3D printed microfluidic device. **A** CAD model of a device with five chambers for direct absorbance measurements on chip. **B** Microfluidic device in a 96-well-plate-adapter for measurement with a plate reader. **C** Test device 3D printed from poly (methyl methacrylate) (PMMA) with a dilution series of dye solution (left to right: 1:1, 1:2, 1:4 and 1:8). Scale bar measures 10 mm. **D** Absorbance spectra of 38.5 µl fluid 2 (same volume as in 3D printed device) in various dilutions (1:1, 1:2, 1:4 and 1:8) in PBS measured in a commercially available 96-well plate. **E** Absorbance spectra of fluid 2 at various dilutions (1:1, 1:2, 1:4 and 1:8) in PBS measured in 3D printed test device. Values are shown as mean ± SD (shown as area) of 3 devices

Direct measurement of absorbance in the microfluidic chip does not require elution, avoiding some of the disadvantages of external measurement, such as dilution or solubility problems. It also simplifies chip design and experimental setup because the entire experiment can be performed on one chip. As shown in Fig. 4, the transparency of the printed device is suitable to perform absorbance measurements with quality comparable to that of commercially available 96-well plates. The slightly higher standard deviation (SD) of the absorbance measurements (Fig. 4E) in the printed chips (SD of A_640nm;fluid 2, 1:1_ to A_640nm; PBS_: 0.0225; 0.0172; 0.0056; 0.0095; 0.0014) compared to the 96-well plate (SD of A_640nm;fluid 2, 1:1_ to A_640nm; PBS_: 0.0070; 0.0079; 0.0045; 0.0019; 0.0012) is likely a result of light scattering at the line-patterned surface. This line-patterned surface topology (Fig. 4C) originates from the manufacturing process of FDM 3D printing and can be improved by post-treatment, for example with solvents. The transparency of the printed microfluidics is sufficient not only to measure absorbances, but also to observe and analyze living cells inside the chip with a microscope (Fig. 6D).

### Biocompatibility of chip material

**Fig. 5:**
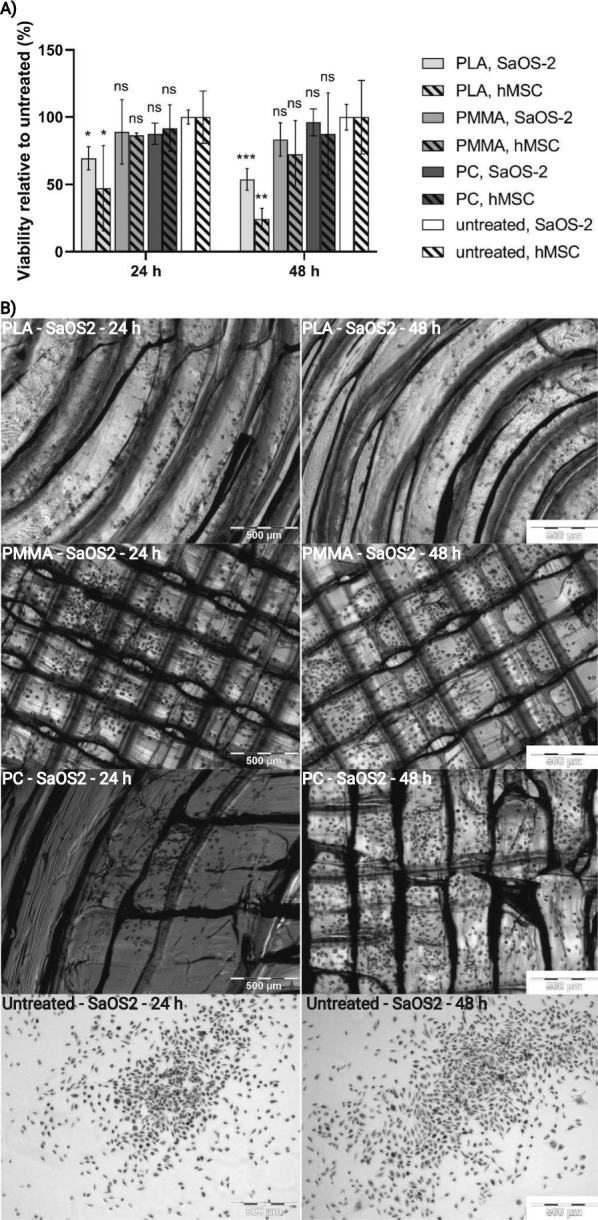
Biocompatibility of FDM 3D-printed polymer discs made of PLA, PMMA and PC was investigated using SaOS-2 osteoblasts and hMSC. **A** The viability of SaOS-2 osteoblasts and hMSC was assessed by MTT assay at 24 and 48 h and compared with the untreated sample (standard 24-well tissue culture plate). Statistical significance was analyzed by Two-way ANOVA and Dunnett's post hoc test compared with the untreated sample (ns, not significant; *p < 0.05; **p < 0.01; ***p < 0.001). **B** Representative microscopic images of FDM printed polymer discs cultivated with SaOS-2 osteoblasts for 24 and 48 h. Images were taken after staining with MTT. Scale bar measures 500 µm. Images were acquired using an Olympus CKX41 (Olympus, Japan) microscope equipped with an Olympus XM10 camera (Olympus, Japan) and associated cellSens Standard software (version 1.9 build 11,514, Olympus, Japan)

For application in biomedical test systems, a biocompatible, non-leaching and non-toxic material is essential for the success of the experiment which disqualifies most commercially available SLA resins [72, 73]. For cell cultures on chip, it is particularly important that no cytotoxic effects occur in direct contact with the material over a period of several days. Therefore, the viability of SaOS-2 osteoblasts and human mesenchymal stem cells (hMSCs) cultured in direct contact with FDM 3D-printed discs made of PLA, PMMA, and PC was investigated. It was observed that the polymers PMMA and PC showed no significant difference in viability to the corresponding cells cultured in tissue culture wells (Fig. 5) after 24 and 48 h. Furthermore, no major morphological changes were detected compared to cells cultured under standard conditions, as shown for the SaOS-2 osteoblasts in Fig. 5B and for the hMSCs in Additional file 1: Fig. S19. The viability of both cell types (SaOS-2 osteoblasts and hMSCs) decreased significantly after being cultured on PLA discs for 24 h. This trend continued for the 48-h samples, resulting in a 46.2 ± 6.6% decrease in SaOS-2 osteoblast viability and a 75.6 ± 6.4% decrease in hMSC viability compared with the respective controls.

### Drug screening system: microfluidic cytotoxicity assay

**Fig. 6:**
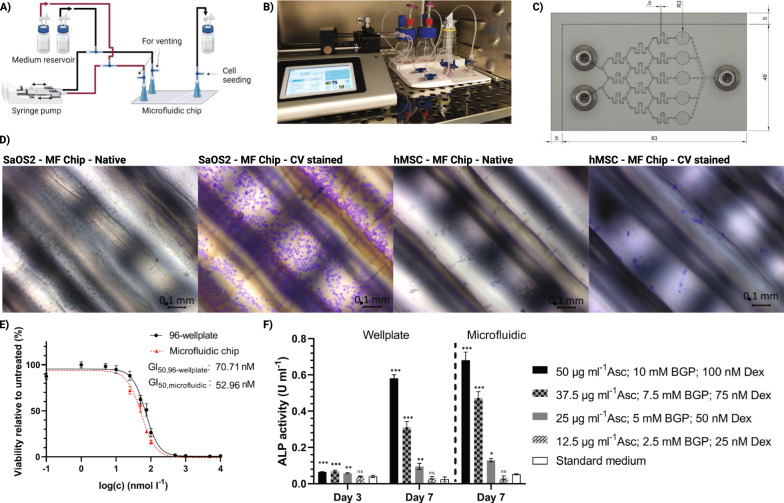
Biological applications: microscopy, growth inhibition (GI) assay and ALP activity assay on 3D printed microfluidic devices (PMMA). **A** Schematic experimental setup of the microfluidic chip with periphery. **B** Photograph of the experimental setup in a humidified incubator. **C** CAD model of a device with five cell culture chambers. **D** Microscopic images of SaOS-2 osteoblasts and hMSCs cultured in the microfluidic (MF) chip for 48 h without additional treatment. Images were taken of native cells and cells stained with 0.1% crystal violet (CV) in PBS. Scale bar measures 100 µm. Images were acquired using an Observer.Z1 (Carl Zeiss AG, Germany) microscope and processed using the software ZEN blue edition (Version 3.4, Carl Zeiss AG, Germany). **E** Comparison of the determined GI50 of staurosporine on SaOS-2 cell viability in a 96-well plate (●) or microfluidic chip (▲) with 150,000 cells cm^−2^. Non-linear regression and GI50 were calculated with GraphPad Prism 8. Values are shown as mean ± SD; 96-well plate (n = 9), microfluidic (3 devices, n = 3). **F** Alkaline phosphatase (ALP) activity of human bone marrow mesenchymal stem cells exposed to different osteogenic supplement concentrations. Values are shown as mean ± SD; 96-well plate (n = 3), microfluidic (3 devices, n = 3). Statistical significance was analyzed with One-way ANOVA and Tukey post-hoc test against standard medium sample (n s, not significant; *p < 0.05; **p < 0.01; ***p < 0.001)

As a first relevant application, we demonstrate a validated cytotoxic screening system that combines a chemical concentration gradient with cell culture chambers and analytics in one chip. Assessment of the half maximal growth-inhibitory concentration (GI_50_) of toxic reagents in a microfluidic chip device resulted in higher reproducibility and accuracy, and less reagent consumption than manual assessment. The superiority of direct 3D printed microfluidic chips was demonstrated by analyzing the GI_50_ value of the potent cytotoxic drug staurosporine (GI_50_ of 13.6 – 105.6 nmol l^−1^, depending on the cell line [74]) on the cell viability of SaOS-2 osteoblasts (Fig. 6E). The calculated GI_50_ values of 52.96 ± 2.43 nmol l^−1^ in the microfluidic chip and 70.71 ± 4.92 nmol l^−1^ in the 96-well plate confirmed the improved reproducibility and accuracy expected from the use of microfluidics for cytotoxicity assays.

### Microfluidic ALP activity assay as an early marker of osteogenic differentiation

As a second relevant application, we demonstrate a validated differentiation system for hMSCs. On the basis of an alkaline phosphatase (ALP) activity assay, a simple microfluidic chip for the cultivation, differentiation and analysis of mesenchymal stem cells into their osteogenic lineage (Fig. 6) was designed. Different concentrations of the osteogenic supplement consisting of ascorbic acid (Asc), β glycerophosphate (BGP) and dexamethasone (Dex) were tested in the microfluidic device and compared to the results obtained via standard cell culture. As shown in Fig. 6F), after 3 days of cultivation, only minor yet significant differences in ALP activity (well plate, standard cell culture) were observed when cells were treated with osteogenic supplement compared with standard medium. On day 7, however, the ALP activity (well plate, standard cell culture) increased substantially in response to the adjusted concentration of osteogenic supplement. The highest concentration of osteogenic supplement (50 µg ml^−1^ Asc, 10 mM BGP and 100 nM Dex) resulted in high ALP activity (0.58 U ml^−1^), whereas with decreasing concentration, the ALP activity (0.58; 0.31; 0.1; 0.03; 0.02 U ml^−1^) also decreased. For the lowest tested concentration (12.5 µg ml^−1^ Asc, 2.5 mM BGP and 25 nM Dex) no significant difference with respect to standard medium without osteogenic supplement was observed. A similar trend was observed for the microfluidic device at day 7, with slightly higher ALP activity (0.68; 0.47; 0.13; 0.03; 0.05 U ml^−1^) than with standard cell culture conditions.

## Discussion

Using a commercial available FDM 3D printer and polymers, we present a method to efficiently manufacture microfluidic chips for biomedical applications. The method presented is cost- and labor-efficient, needs little prior knowledge, avoids toxic or leaching chemicals, allows easy adaptation of the chip to the specific challenges of the experiment and produces ready-to-use as well as long-term biocompatible microfluidics. In addition, a 3D chip design can be produced, offering new possibilities compared to traditional manufacturing methods that can only create 2D chip designs.

The observed difference between printed test objects and the CAD model for the achieved resolution (Fig. 2) can partially be explained by the accuracy of the printer´s step motors, which the manufacturer indicates is ± 12.5 µm [75]. In addition, the printing process itself can explain the deviation. During the printing process, the polymer melts in the print head and is extruded through the nozzle. Subsequently, the polymer cools and becomes solid again, forming a unit with the surrounding material. A short cooling time is crucial to achieving high resolution, because as long as the polymer is in its fluid state, external forces such as gravity or vibrations alter its final position in the object. This phenomenon has been observed particularly for PC, which requires cooling fan deactivation and a high ϑ during the printing process; otherwise the polymer leaving the nozzle does not attach to the previously printed layer. This problem can be remedied by using a closed print chamber with controlled ambient temperature by raising the temperature of the previously printed layers [76]. Consequently, the adhesion of the subsequent layer is improved and the cooling fan can be activated to dissipate the heat emitted by the printhead. Since we wanted to demonstrate the feasibility of producing microfluidic devices using a commercially available FDM 3D printer without any customizations, we did not test a heated print chamber in this study. The effects of this phenomenon can be clearly observed in chips made from PC (Fig. 3). The corners of the channels were narrower than those in the CAD model, and the straight channels between them had a slightly oval shape. This had a particularly strong impact on the performance of the mixer design, where slight fluctuations in the channel widths decreased or increased the hydrodynamic resistance (R_H_); R_H_ is anti-proportional to the circular channel radius (r) and increases with channel length (L) and dynamic viscosity (µ), as shown in Eq. 1 [77]. Thus, channels with a greater diameter show less hydrodynamic resistance, which results in higher flow rates (Q) (Eq. 2), since the pressure difference ($\mathrm{\Delta p}$) remains constant. Consequently, fluctuations in Q observed at each channel branch ultimately influence the volume fractions at the chips’ outlets.

1 $${R}_{H}=\frac{8\upmu L}{\pi {r}^{4}}$$

2 $$\mathrm{\Delta p }=\mathrm{ Q}*{R}_{H}$$

As listed in Table 1, the polymers tested have different advantages and disadvantages and find different applications accordingly. It is worth mentioning that PLA is hydroscopic and the dimensions of the channels may change over time due to swelling when used in contact with water. Especially at very low flow rates, such as in a long-term cell culture, the change in channel dimensions or the lactic acid released by the degradation can have a negative effect on the experiment. Furthermore, swelling itself can also lead to differences between the effective concentration (c_eff_) and the set concentrations (c_set_). For this reason, we would recommend PLA for the development of “quick and dirty” prototypes or for experiments with a short duration, since the printing properties are excellent and the effects mentioned above are mainly seen in longer experiments. In longer experiments, PMMA would be more suitable as a chip material, as it does not have the problems mentioned for PLA, but at the same time has very good printing properties and biocompatibility. With PC, on the other hand, the inaccuracy during the printing process must be taken into account if a heatable printing chamber is not available. Nevertheless, PC is recommended for experiments requiring higher temperatures than 90 °C, such as PCR applications, especially if the chip has only one channel, as it is form-stable even at temperatures up to 140 °C. It should also be mentioned that when solvents are used, care should be taken to ensure that they do not attack the polymer in question.

Additionally, our observations confirm the advantages of FDM 3D printing for biomedical applications, substantially by a wide range of commercially available long-term biocompatible materials. Zhu et al. [78] tested several materials printed with FDM, Multi-Jet Modelling, and SLA and observed high toxicity for several species, except for the samples printed with FDM. This was one of the reasons why we decided against SLA 3D printing and in favor of FDM 3D printing, despite the better resolution of SLA [79]. The biocompatibility of PMMA and PC, as reported in the literature [48, 49, 80], can be confirmed by the results obtained in this study. In contrast, more ambivalent findings are described in the literature for PLA. For example, Li et al. [81] and Silva et al. [82] observed good biocompatibility, while Lee et al. [80] reported inflammatory responses to PLA scaffolds and Majidi et al. [83] observed a reduction in viability of L929 fibroblast cells by almost 50% after 72 h, although they attributed this to reduced cell attachment and not a toxic effect. In agreement with the results of Majidi et al., we observed a decrease in cell viability for PLA, which can also be attributed to either decreased cell adhesion or a toxic effect. In any case, the tested PLA is not suitable for use in microfluidic devices with biological applications without further treatment. The PLA used is of technical grade and contains different additives depending on the manufacturer, so it may vary from manufacturer to manufacturer. This shows that it is necessary and useful to test polymers for cytotoxicity before their application in biological systems, thus avoiding side effects or misleading results.

Based on the properties (print resolution, optical transparency, and biocompatibility) of the polymers tested, we selected PMMA as the polymer of choice for the subsequent microfluidic devices with biomedical applications. We observed better performance for the microfluidic chips than the 96-well plate experiments, both for the cytotoxic screening system and the osteogenic differentiation system. The observed lower GI_50_ for the microfluidic cytotoxic screening system compared with the 96-well plate could be due to two effects: first, the concentration settings in the microfluidic chip might have been more accurate than manual pipetting, and consequently, the lower scatter in the measurement data decreased the likelihood of statistical outliers being included in the calculation. Second, the dynamic cultivation in the microfluidic system might have ensured constant and uniformly distributed concentrations in the cultivation chambers, such that c_set_ corresponded to c_eff_ within the cells. In contrast, lower concentrations might have occurred locally in the static system of a 96-well plate, thus resulting in a lower c_eff_. Another advantage of the microfluidic system is its faster preparation time, particularly when the same assay is performed several times, for example, in a routine analysis or a high-throughput experiment.

The better performance of the microfluidic assay for osteogenic differentiation was reflected in increased ALP activity and demonstrated the importance of in vivo-mimicking conditions. This might have been a result of synergistic effects of the shear stress, which is constantly present in the dynamic culture system of the microfluidic device [84] and is known to have a positive effect on osteogenic differentiation of mesenchymal stem cells [85–88]. With this simple microfluidic chip, the osteogenic effect of supplements at different concentrations was successfully confirmed after 7 days of cultivation without the need for extensive manual work. Furthermore, the chip in combination with the ALP activity assay could be used to analyze the osteogenic effects of several other chemicals or could be combined with other colorimetric assays or fluorescent probes to analyze different cellular functions. Both applications can be further improved by adding additional chambers to the chip design, which can reduce concentration intervals or cover a wider concentration range. In addition, testing combinations of several compounds should be possible by creating a 3D gradient generator that can accommodate four or even more inlets.

## Conclusions

In summary, microfluidic methods offer many advantages over current standard methods, especially in dynamic cell culture systems, but are partially limited in their biocompatibility, availability and adaptability. To provide a solution to this problem, we present the use of a conventional, unmodified 3D printer for the cost-efficient and rapid production of customizable and biocompatible microfluidic chips. We demonstrated the suitability of 3D printing for reproducible production of 100 µm channel structures for 2D and 3D chip structures and layouts. In addition, we demonstrated the applicability and superiority of self-printed microfluidic chips in cell culture, both in assessing cytotoxicity, and in inducing and analyzing stem cell differentiation in dynamic culture systems. The chips presented have the advantages that all steps of an experiment (preparation/treatment, cultivation, analysis) are performed on one chip and can be easily adapted to the specific challenges of the experimental design. This can be realized, for example, by increasing the number of cell culture chambers for higher data density, integrating sensor systems into the chip design, adding new inlets for feeding additional chemicals, or using a different chip material for surface modification or temperature optimization.

## Materials and methods

Chips were printed with an Ultimaker 3 (Ultimaker, Netherlands) FDM 3D printer with a 0.4 mm nozzle head using 2.85 mm polymer filaments purchased from filamentworld.com (Germany). Polymers were purchased in transparent forms: PLA-transparent (PLA300XCLR), PMMA-transparent (PMM300XCLR) and PC-transparent (PCA300XCLR). To validate and apply the created microfluidic devices, we used a Legato 111 (KD Scientific, United States of America) syringe pump. The test fluids were a mixture of water and food coloring (Ruf, Germany) purchased from a local store. Blue solution (fluid 2) was adjusted to an A_640nm_ of 1, and red solution (fluid 1) was adjusted to match the viscosity of the blue solution, thus resulting in an A_490nm_ of 0.45. Absorbance measurements were performed with a TECAN infinite M200 PRO (TECAN, Switzerland) plate reader. SaOS-2 human osteogenic sarcoma cells (ACC 243, DSMZ) were cultured in McCoy’s 5A medium supplemented with 10% fetal calf serum, penicillin at 100 U ml^−1^ and streptomycin at 100 U ml^−1^. Human bone marrow derived mesenchymal stem cells (hMSC) were cultured in stem cell expansion medium SCM015 supplemented with penicillin at 100 U ml^−1^ and streptomycin at 100 U ml^−1^. Chemicals and cell culture media were purchased from Sigma Aldrich, Germany, unless stated otherwise.

### Fabrication of devices

An overview of the manufacturing process of microfluidic chips with a 3D printer is shown in Fig. 1. First, a CAD model was created in Autodesk Fusion 360 (Autodesk, USA). The model included the structures contained in the finished chip, such as channel structures, reaction chambers and tube connections. Then the CAD model was exported as an STL file and uploaded to the open source slicer software Cura (Version 4.6.1, Ultimaker, Netherlands). In this step, users can change various parameters affecting the printing process. By adjusting these parameters to the polymer used or the structures to be printed, the printing results can be improved. After the CAD model is sliced according to the entered parameters, the software saves the information in a G-code file that is sent to the 3D printer. After 30 to 60 min, depending on the chip size, the printing process is complete, and the device can be removed from the print bed. It can be used immediately or further modified for complex applications. The Cura files with the adjusted slicing parameters for the three listed polymers are provided in the Additional file 2.

### Resolution assessment of direct 3D printed microfluidic channels

Test specimens with channels in the X-, Y- and Z-directions were printed to study the influence of different parameters on the print resolution. The parameters analyzed were printing temperature (ϑ), printing speed (v), layer height (h) and fan speed (fan). Images of the channels in the X-, Y- and Z-directions were taken from the printed test device by using an Olympus CKX41 (Olympus, Japan) microscope with a mounted Olympus XM10 camera (Olympus, Japan) and the associated software cellSens Standard (Version 1.9 Build 11,514, Olympus, Japan). Subsequently, the achieved channel widths and heights were calculated with imageJ (Version 1.52a, National Institutes of Health, USA) and compared with the given values of the CAD model. In addition, a visual inspection was performed after injection of liquid into the channels to ensure that they were not blocked.

### 3D microfluidic structures

Microfluidic devices were fabricated for each of the three polymers (PLA, PMMA and PC). Chips were tested for leakage and functionality by injection of test liquids. Several chip components were generated, which can be arranged and combined on a chip depending on the application. Three chips were designed, containing structures of varying complexity. The first was a chip with two intersecting serpentine channels with a spacing in the Z-direction of 0.2 mm. This chip demonstrated the printability of multiple structures on top of each other in one device without leakage. The second was a chip with two straight channels crossing each other with a bridge, which demonstrated the generation of channels not only in the X- and Y-directions, but also in the Z-direction. The third was a chip containing spirals of different diameters and cross-section geometries, which are representative of the implementation of complex 3D structures in the chip design.

### Microfluidic gradient generator

Microfluidic mixer components are used in many microfluidic chips. These structures are primarily used to mix multiple liquid streams but can also be used to create a concentration gradient across different liquid streams with the correct arrangement of microfluidic channels. The gradient generator chip that we fabricated included two inlets, five outlets and all channels of the same sizes, thus resulting in the theoretical volume fractions listed in Table 3. The theoretical fluid compositions at the outlets were verified by injecting two solutions, red solution (fluid 1) and blue solution (fluid 2), into the microfluidic devices with a Legato 111 (KD Scientific, Germany) syringe pump. Each inlet was adjusted to a flow of 0.2 ml min^ −1^. After equilibrium was reached, images of the chip were collected, and the outlets were emptied. A 100 µl volume of each of the fluids collected from the outlets was transferred to a 96-well plate, and the absorbance at 640 nm and 490 nm was measured with a plate reader. The proportion of blue and red fluid was calculated according to a linear calibration curve.

**Table 3:** Theoretical volume fractions of fluids entering or leaving the passive microfluidic mixer

	Inlet 1	Inlet 2	Outlet 1	Outlet 2	Outlet 3	Outlet 4	Outlet 5
Theoretical volume fraction of fluid 1 (φ_fluid1_)^a^	1	0	1	0.75	0.5	0.25	0
Theoretical volume fraction of fluid 2 (φ_fluid2_)^b^	0	1	0	0.25	0.5	0.75	1

^a^red solution (fluid 1)

^b^blue solution (fluid 2)

### Absorbance measurement on chip

A microfluidic chip with five round chambers (diameter = 7 mm, height = 1 mm) was generated. The 96-well plate format was chosen to allow the chip to be measured in a standard plate reader. For this purpose, the chip was plugged into a 3D printed adapter and measured analogously to a conventional 96-well plate. First, every chamber was filled with PBS, and the absorbance spectra were measured as the offset value. Afterward, PBS was replaced by a dilution series of test solutions (1:1, 1:2, 1:4 and 1:8 of fluid 2 in PBS). The absorbance spectra were measured and compared with the absorbance spectra in a conventional 96-well plate.

### Biocompatibility of chip material

Biocompatibility analyses were performed with SaOS-2 human osteogenic sarcoma cells and hMSC in direct contact with the polymers PLA, PMMA and PC. 3D-printed polymer discs (r = 6.5 mm, h = 0.2 mm) were seeded with 50,000 cells each and cell viability was assessed after 24 and 48 h by 3-(4,5-dimethylthiazol-2-yl)-2,5-diphenyltetrazolium bromide (MTT) assay. Briefly, polymer discs were placed in a 24-well plate and a cell suspension containing 5 × 10^6^ cells ml^−1^ was prepared (hMSCs were used at passage 2). 10 µl of the cell suspension was dispersed on the polymer discs and incubated at 37 °C and 5% CO_2_. After 30 min, 50 µl of medium was carefully added to prevent drying, and after another 3.5 h, 500 µl of medium was added. The medium was replaced by 500 µl MTT solution (MTT at 1 mg ml^−1^ in medium) at cultivation times of 24 or 48 h and samples were incubated for 2 h. Afterwards, polymer discs were washed 2 times with 500 µl PBS and transferred to a new well. Formazan crystals were dissolved by adding 500 µl of DMSO and quantified in triplicate á 100 µl at 570 nm in a plate reader. Cell viability was calculated as the ratio of absorbance between cells cultured on polymer discs and cells cultured in a tissue culture well.

### Microfluidic cytotoxicity assay

SaOS-2 human osteogenic sarcoma cells were used to determine the half maximal growth-inhibitory concentration (GI_50_) of staurosporine (APExBIO Technology LLC, USA). Cells were seeded on the microfluidic chip at a density of 150,000 cells cm^−2^ after the chip had been filled with sterile PBS. The seeding process was performed as follows: the cell chamber was filled with cell suspension via the chip outlet using a pipette. The filling was stopped as soon as the cell chamber was completely filled to ensure that no cells adhered in the channels of the upstream. The fill level was visually determined by the color difference between cell suspension (red) and sterile PBS (transparent). Before treatment with staurosporine at 0, 25, 50, 75 and 100 nmol l^−1^, cells were allowed to attach to the cell culture chambers for 4 h. Excess cells adhering in the downstream channels are partially flushed out of the system by the applied flow of the experiment. Treatment was performed with a flow rate of 0.008 ml h^−1^ per cell culture chamber by injection of culture medium (inlet 1; 0.02 ml h^−1^) and staurosporine at 100 nmol l^−1^ in culture medium (inlet 2; 0.02 ml h^−1^). In addition, SaOS-2 cells (150,000 cells cm^-2^) were treated with 100 µl medium supplemented with staurosporine at 0, 1, 5, 10, 25, 50, 75, 100, 500, 1,000, 5,000 and 10,000 nmol l^−1^ in 96-well culture plates by manual pipetting. Afterwards, the cells were incubated in a humidified incubator at 37 °C and 5% CO_2_ for 24 h before cell viability was assessed with MTT assay. First, the medium was replaced with 100 µl MTT solution (MTT at 1 mg ml^−1^ in medium) and incubated for 2 h. Subsequently, the MTT solution was removed, and 100 µl 10 (w/v)% sodium dodecyl sulfate in phosphate-buffered saline (PBS) was added and incubated for another 4 h. The absorbance was measured at 570 nm directly in the chip and the 96-well plate by using a plate reader. Cell viability was calculated as the ratio of absorbance between the samples with and without staurosporine treatment. MTT assays were repeated three times, each time with a new device, and the GI_50_ value was calculated with GraphPad Prism 8 (GraphPad Software, USA).

### Microfluidic ALP activity assay as an early marker of osteogenic differentiation

Alkaline phosphatase (ALP) activity was assessed in a microfluidic chip as an early marker of the osteogenic differentiation of hMSCs. Cells were cultured in stem cell expansion medium SCM015 (Sigma Aldrich, Germany) supplemented with penicillin at 100 U ml^−1^, streptomycin at 100 U ml^−1^ and different concentrations of osteogenic supplement (Asc, BGP and Dex) that has been shown in the literature to induce osteogenic differentiation [89, 90]. At passage three, hMSCs were seeded at a density of 150,000 cells cm^−2^ in the microfluidic chip as well as in a 96-well culture plate, as previously described, and treated with osteogenic supplement for 7 days in a humidified incubator at 37 °C and 5% CO_2_. Dilution of the osteogenic supplement stock (Asc at 500 µg ml^−1^ (Carl Roth, Germany), BGP at 100 mmol l^−1^ (Carl Roth, Germany) and Dex at 1 µmol l^−1^) was performed in medium, thus resulting in 10, 7.5, 5 and 2.5 (v/v)% stock concentrations for the 96-well plate. SCM015 without osteogenic supplement was used as a control. The microfluidic chip was injected with SCM015 and 10 (v/v)% stock concentrations, each at one inlet, thus resulting in theoretical concentrations of 10, 7.5, 5, 2.5 and 0 (v/v)% stock concentrations in the cell chambers. ALP activity was assessed with an Alkaline Phosphatase Diethanolamine Activity Kit (AP0100, Sigma Aldrich, Germany) according to the manufacturer’s instructions with modifications. Briefly, after cultivation, the medium was replaced with 100 µl reaction solution consisting of 98 µl reaction buffer (included in the kit) with 1(v/v)% Triton X-100 and 2 µl 0.67 mol l^−1^ p-nitrophenyl phosphate (included in the kit). The absorbance was measured every 30 s for 300 s at 405 nm directly in the chip or the 96-well plate with a plate reader. The average linear rate (A_405nm_ min^−1^) was used to calculate the ALP activity according to a calibration curve.

### Numeric simulation of fluidic behavior in microfluidic chips

Numeric simulation was performed with the finite element method (FEM) to verify the observed mixing ratios in the designed microfluidic chips. For this purpose, a system consisting of two phases of water was defined. Boundary conditions for the inlets were set according to the experiments. The outlet boundary condition was set to a pressure outlet with a gauge pressure of 0 Pa. The simulation was performed with ANSYS 2020 R1 Academic (ANSYS Inc., United States of America) by using the simplec algorithm. The volume fractions of both phases were calculated and displayed along the longitudinal section.

## Supplementary Information

**Additional file 1: Figure S1.** Obtained dimensions of the 3D printed poly (lactic acid) (PLA) test device for various printing temperatures. Remaining parameters were kept constant at v = 30 mm/s, h = 50 µm and fan = 100%. Results are shown for the X-, Y- and Z-axis separately. Values shown as mean ± standard deviation of 3 devices. **Figure S2.** Obtained dimensions of the 3D printed poly (methyl methacrylate) (PMMA) test device for various printing temperatures. Remaining parameters were kept constant at v = 50 mm/s, h = 100 µm and fan = 50%. Results are shown for the X-, Y- and Z-axis separately. Values shown as mean ± standard deviation of 3 devices. **Figure S3.** Obtained dimensions of the 3D printed polycarbonate (PC) test device for various printing temperatures. Remaining parameters were kept constant at v = 50 mm/s, h = 100 µm and fan = 0%. Results are shown for the X-, Y- and Z-axis separately. Values shown as mean ± standard deviation of 3 devices. **Figure S4.** Obtained dimensions of the 3D printed poly (lactic acid) (PLA) test device for various printing speeds. Remaining parameters were kept constant at ϑ = 190 °C, h = 50 µm and fan = 100%. Results are shown for the X-, Y- and Z-axis separately. Values shown as mean ± standard deviation of 3 devices. **Figure S5.** Obtained dimensions of the 3D printed poly (methyl methacrylate) (PMMA) test device for various printing speeds. Remaining parameters were kept constant at ϑ = 245 °C, h = 100 µm and fan = 50%. Results are shown for the X-, Y- and Z-axis separately. Values shown as mean ± standard deviation of 3 devices. **Figure S6.** Obtained dimensions of the 3D printed polycarbonate (PC) test device for various printing speeds. Remaining parameters were kept constant at ϑ = 240 °C, h = 100 µm and fan = 0%. Results are shown for the X-, Y- and Z-axis separately Values shown as mean ± standard deviation of 3 devices. **Figure S7.** Obtained dimensions of the 3D printed poly (lactic acid) (PLA) test device for various layer heights. Remaining parameters were kept constant at ϑ = 190 °C, v =70mm/sand fan = 100%. Results are shown for the X-, Y- and Z-axis separately. Values shown as mean ± standard deviation of 3 devices. **Figure S8.** Obtained dimensions of the 3D printed poly (methyl methacrylate) (PMMA) test device for various layer heights. Remaining parameters were kept constant at ϑ = 245 °C, v = 70 mm/s and fan = 50%. Results are shown for the X-, Y- and Z-axis separately. Values shown as mean ± standard deviation of 3 devices. **Figure S9.** Obtained dimensions of the 3D printed polycarbonate (PC) test device for various layer heights. Remaining parameters were kept constant at ϑ = 240 °C, v = 80 mm/s and fan = 0%. Results are shown for the X-, Y- and Z-axis separately. Values shown as mean ± standard deviation of 3 devices. **Figure S10. **Obtained dimensions of the 3D printed poly (lactic acid) (PLA) test device for various fan speeds. Remaining parameters were kept constant at ϑ = 190 °C, v = 70 mm/s and h = 100 µm. Results are shown for the X-, Y- and Z-axis separately. Values shown as mean ± standard deviation of 3 devices. **Figure S11.** Obtained dimensions of the 3D printed poly (methyl methacrylate) (PMMA) test device for various fan speeds. Remaining parameters were kept constant at ϑ = 245 °C, v = 70 mm/s and h = 100 µm. Results are shown for the X-, Y- and Z-axis separately Values shown as mean ± standard deviation of 3 devices. **Figure S12.** Obtained dimensions of the 3D printed polycarbonate (PC) test device for various fan speeds. Remaining parameters were kept constant at ϑ = 240 °C, v = 80 mm/s and h = 100 µm. Results are shown for the X-, Y- and Z-axis separately. Values shown as mean ± standard deviation of 3 devices. **Figure S13.** Representative microscopic images of FDM printed micro channels in a poly (lactic acid) (PLA) test device. Analysis performed with “imageJ” (Version 1.52a, National Institutes of Health, USA). Scale bar measures 200 µm. **Figure S14.** Representative microscopic images of FDM printed micro channels in a poly (methyl methacrylate) (PMMA) test device. Analysis performed with “imageJ” (Version 1.52a, National Institutes of Health, USA). Scale bar measures 200 µm. **Figure S15.** Representative microscopic images of FDM printed micro channels in a PC test device. Analysis performed with “imageJ” (Version 1.52a, National Institutes of Health, USA). Scale bar measures 200 µm. **Figure S16.** Relative deviation between experimental dimensions and CAD dimensions in X-direction. **Figure S17.** Relative deviation between experimental dimensions and CAD dimensions in X-direction. **Figure S18.** Relative deviation between experimental dimensions and CAD dimensions in X-direction. **Figure S19.** Representative microscopic images of FDM printed polymer discs cultivated with human mesenchymal stem cells (hMSC) for 24 and 48 hours. Viability of hMSC cultivated on PLA, PMMA and PC discs was analyzed with MTT assay and compared to untreated hMSCs cultivated in a standard tissue culture 24-well plate. Images were taken after staining with MTT. Scale bar measures 500 µm.

**Additional file 2**. PLA.curaprofile contains the optimized slicing parameters for the polymer PLA, which can be imported into the slicing program Cura. PMMA.curaprofile contains the optimized slicing parameters for the polymer PMMA, which can be imported into the slicing program Cura. PC.curaprofile contains the optimized slicing parameters for the polymer PC, which can be imported into the slicing program Cura.

## Data Availability

All data generated or analysed during this study are included in this published article [and its Additional information files].
