# Supplementary material for: Direct 3D printed biocompatible microfluidics: assessment of human mesenchymal stem cell differentiation and cytotoxic drug screening in a dynamic culture system
Source: J Nanobiotechnology. 2022 Dec 27;20:540. doi: 10.1186/s12951-022-01737-7 (PMC9793564; doi:10.1186/s12951-022-01737-7)
Supplement: Supplementary file 1 — Additional file 1: Figure S1. Obtained dimensions of the 3D printed poly (lactic acid) (PLA) test device for various printing temperatures. Remaining parameters were kept constant at v = 30 mm/s, h = 50 µm and fan = 100%. Results are shown for the X-, Y- and Z-axis separately. Values shown as mean ± standard deviation of 3 devices. Figure S2. Obtained dimensions of the 3D printed poly (methyl methacrylate) (PMMA) test device for various printing temperatures. Remaining parameters were kept constant at v = 50 mm/s, h = 100 µm and fan = 50%. Results are shown for the X-, Y- and Z-axis separately. Values shown as mean ± standard deviation of 3 devices. Figure S3. Obtained dimensions of the 3D printed polycarbonate (PC) test device for various printing temperatures. Remaining parameters were kept constant at v = 50 mm/s, h = 100 µm and fan = 0%. Results are shown for the X-, Y- and Z-axis separately. Values shown as mean ± standard deviation of 3 devices. Figure S4. Obtained dimensions of the 3D printed poly (lactic acid) (PLA) test device for various printing speeds. Remaining parameters were kept constant at ϑ = 190 °C, h = 50 µm and fan = 100%. Results are shown for the X-, Y- and Z-axis separately. Values shown as mean ± standard deviation of 3 devices. Figure S5. Obtained dimensions of the 3D printed poly (methyl methacrylate) (PMMA) test device for various printing speeds. Remaining parameters were kept constant at ϑ = 245 °C, h = 100 µm and fan = 50%. Results are shown for the X-, Y- and Z-axis separately. Values shown as mean ± standard deviation of 3 devices. Figure S6. Obtained dimensions of the 3D printed polycarbonate (PC) test device for various printing speeds. Remaining parameters were kept constant at ϑ = 240 °C, h = 100 µm and fan = 0%. Results are shown for the X-, Y- and Z-axis separately Values shown as mean ± standard deviation of 3 devices. Figure S7. Obtained dimensions of the 3D printed poly (lactic acid) (PLA) test device for various layer heigh [file 12951_2022_1737_MOESM1_ESM.docx]

**Direct 3D printed biocompatible microfluidics: assessment of human mesenchymal stem cell differentiation and cytotoxic drug screening in a dynamic culture system**

**Oliver Riester ^1,2^, Stefan Laufer ^2,3^ and Hans-Peter Deigner ^*,1,4,5^**

^1^ Institute of Precision Medicine, Furtwangen University, Jakob-Kienzle-Strasse 17, Villingen-Schwenningen, 78054, Germany

^2^ Institute of Pharmaceutical Sciences, Department of Pharmacy and Biochemistry, Eberhard-Karls-University Tuebingen, Auf der Morgenstelle 8, Tuebingen, 72076, Germany

^3^ Tuebingen Center for Academic Drug Discovery & Development (TüCAD2), 72076 Tuebingen, Germany

^4^ Faculty of Science, Eberhard-Karls-University Tuebingen, Auf der Morgenstelle 8, Tuebingen, 72076, Germany

^5^ EXIM Department, Fraunhofer Institute IZI (Leipzig), Schillingallee 68, Rostock, 18057, Germany

Influence of printing parameters

The influence of the parameters, printing temperature (ϑ), printing speed (v), layer height (h) and fan speed (fan) on the achieved printing resolution was analyzed. The following diagrams show the results for each tested polymer and parameter. The original parameters are based on the manufacturer's specifications for the respective polymer.

Analyzed parameter: printing temperature (ϑ)


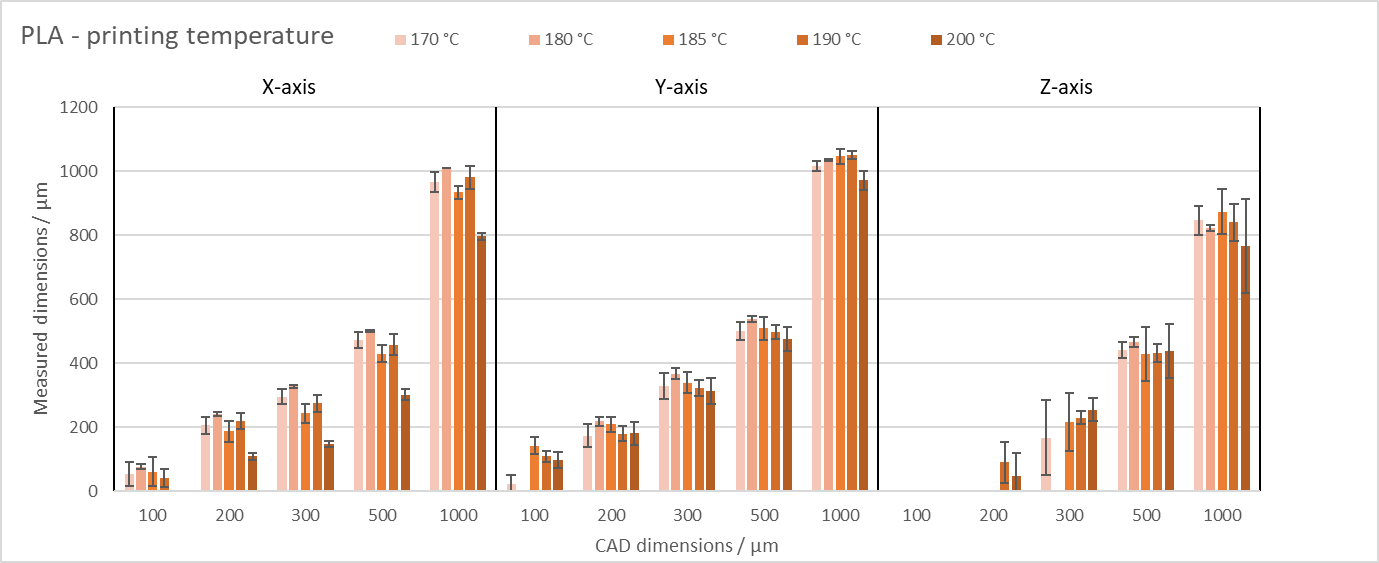


**Fig. S1|Obtained dimensions of the 3D printed poly (lactic acid) (PLA) test device for various printing temperatures.** Remaining parameters were kept constant at v = 30 mm/s, h = 50 µm and fan = 100%. Results are shown for the X-, Y- and Z-axis separately. Values shown as mean ± standard deviation of 3 devices.


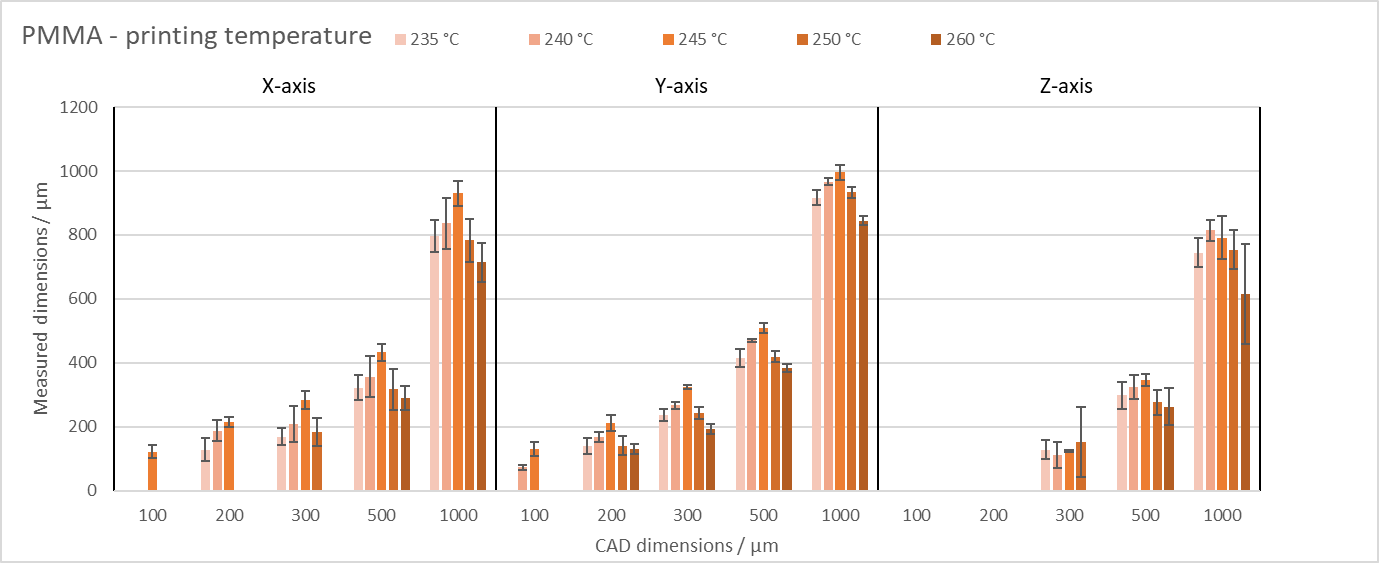


**Fig. S*2* | Obtained dimensions of the 3D printed poly (methyl methacrylate) (PMMA) test device for various printing temperatures.** Remaining parameters were kept constant at v = 50 mm/s, h = 100 µm and fan = 50%. Results are shown for the X-, Y- and Z-axis separately. Values shown as mean ± standard deviation of 3 devices.


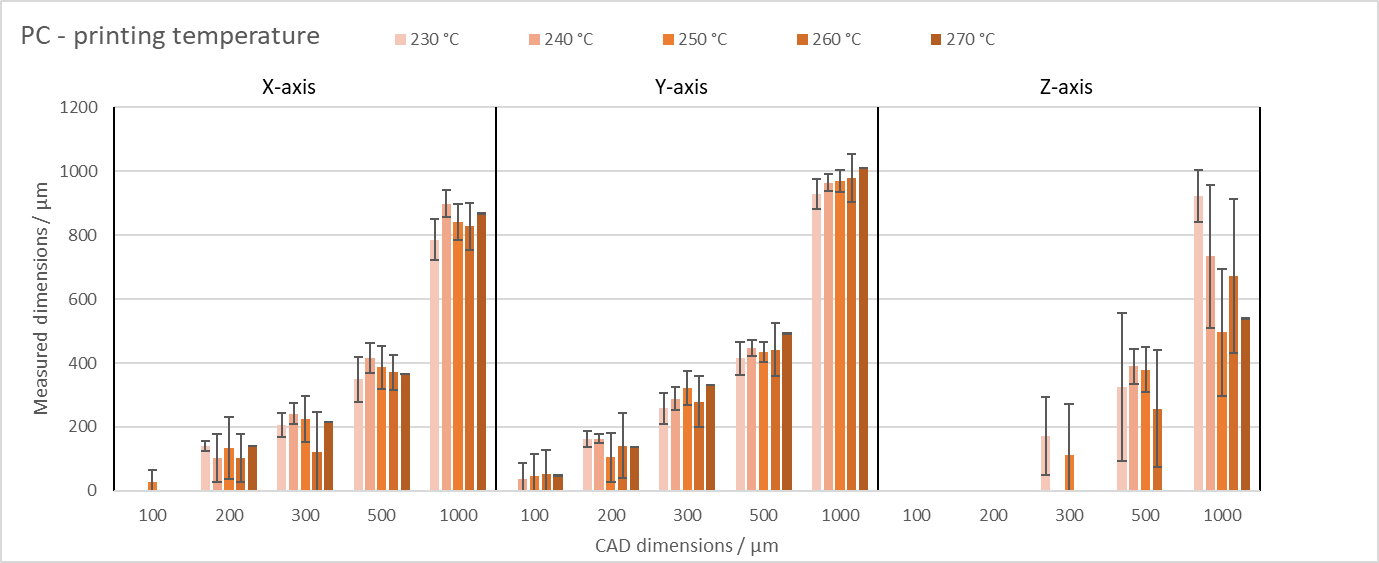


**Fig. S*3* |Obtained dimensions of the 3D printed polycarbonate (PC) test device for various printing temperatures.** Remaining parameters were kept constant at v = 50 mm/s, h = 100 µm and fan = 0%. Results are shown for the X-, Y- and Z-axis separately. Values shown as mean ± standard deviation of 3 devices.

Analyzed parameter: printing speed (v)


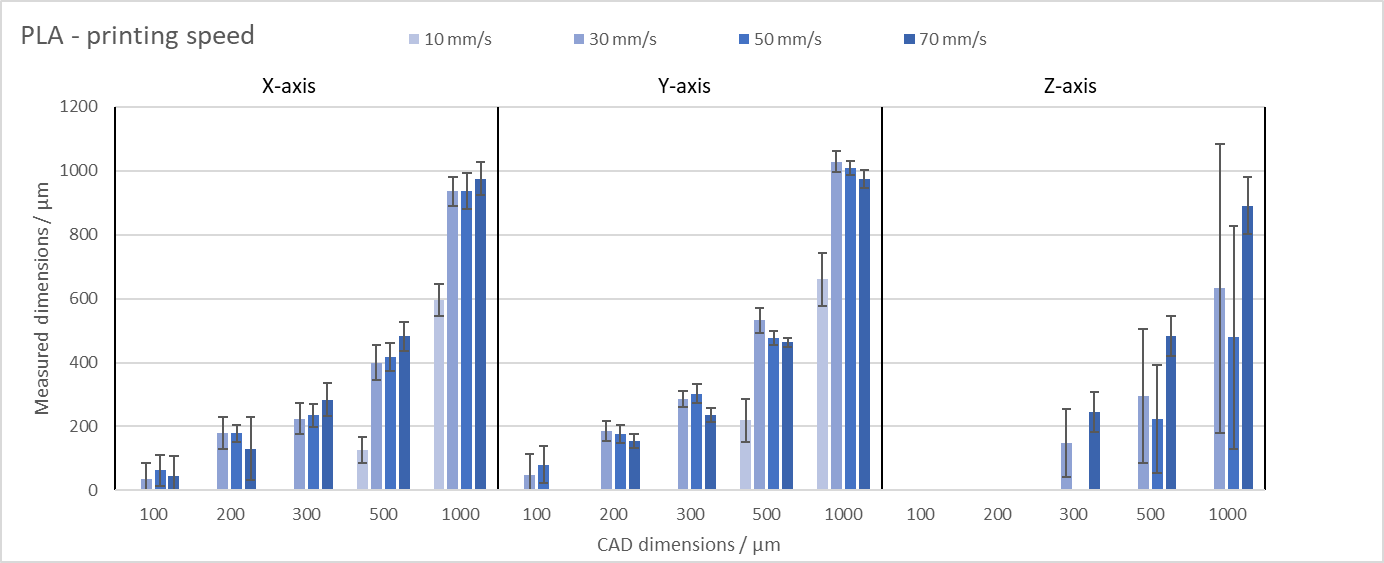


**Fig. S4|Obtained dimensions of the 3D printed poly (lactic acid) (PLA) test device for various printing speeds.** Remaining parameters were kept constant at ϑ = 190 °C, h = 50 µm and fan = 100%. Results are shown for the X-, Y- and Z-axis separately. Values shown as mean ± standard deviation of 3 devices.


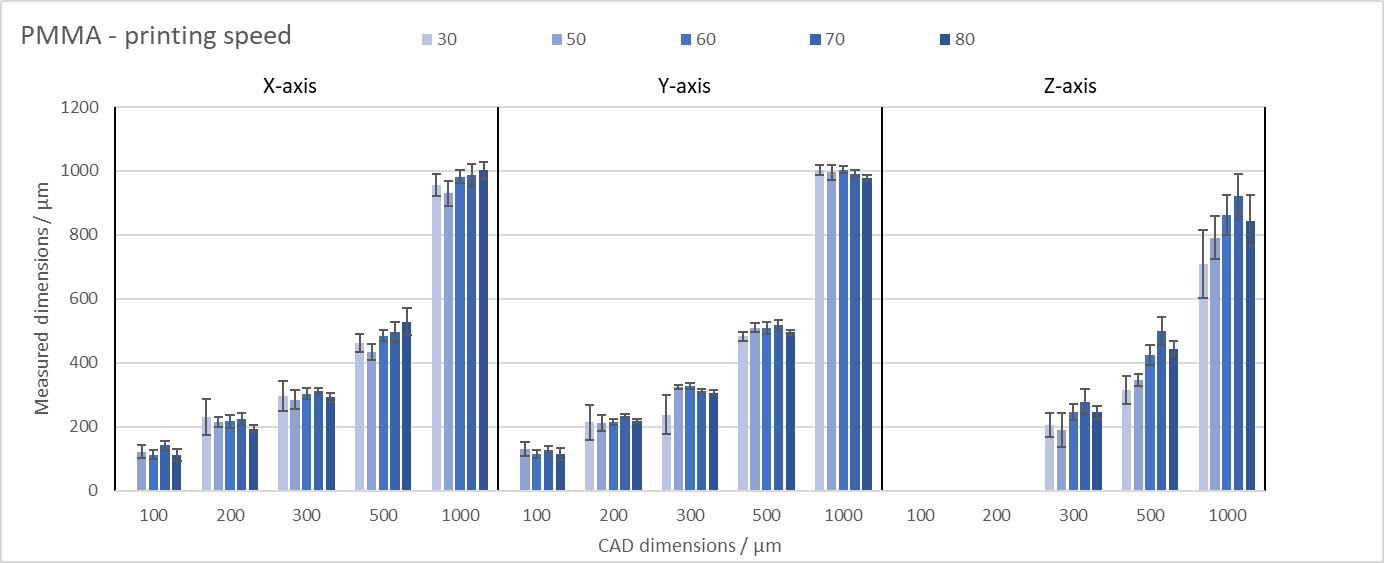


**Fig. S*5* |Obtained dimensions of the 3D printed poly (methyl methacrylate) (PMMA) test device for various printing speeds.** Remaining parameters were kept constant at ϑ = 245 °C, h = 100 µm and fan = 50%. Results are shown for the X-, Y- and Z-axis separately. Values shown as mean ± standard deviation of 3 devices.


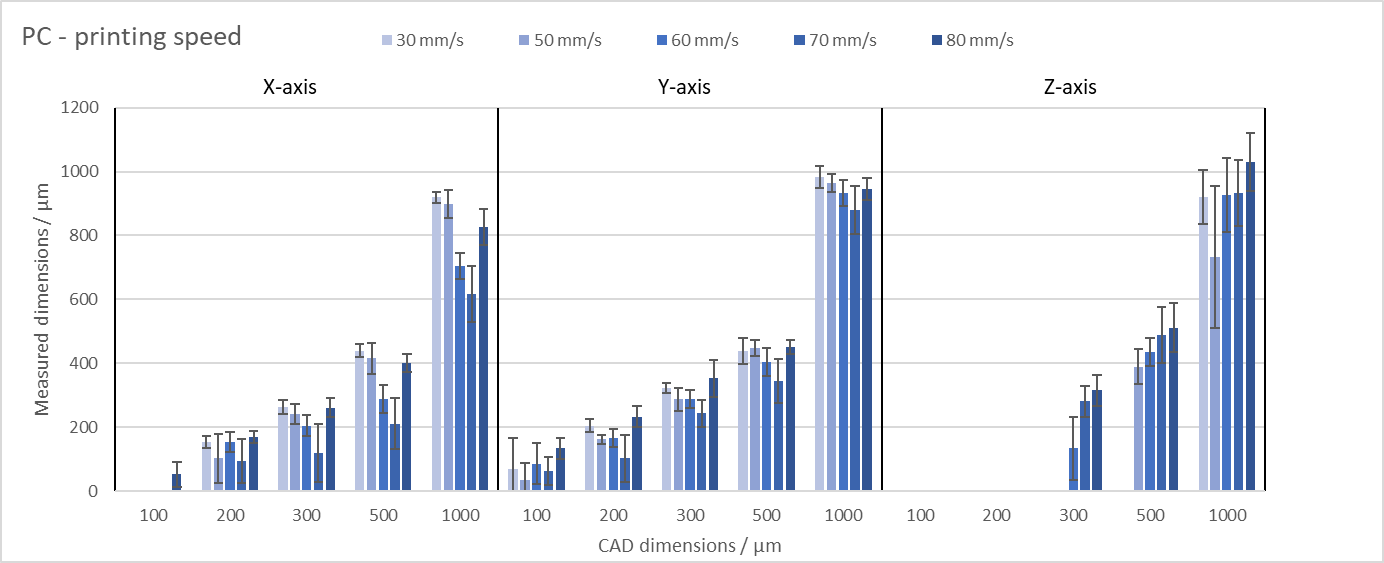


**Fig. S6| Obtained dimensions of the 3D printed polycarbonate (PC) test device for various printing speeds.** Remaining parameters were kept constant at ϑ = 240 °C, h = 100 µm and fan = 0%. Results are shown for the X-, Y- and Z-axis separately Values shown as mean ± standard deviation of 3 devices.

Analyzed parameter: layer height (h)


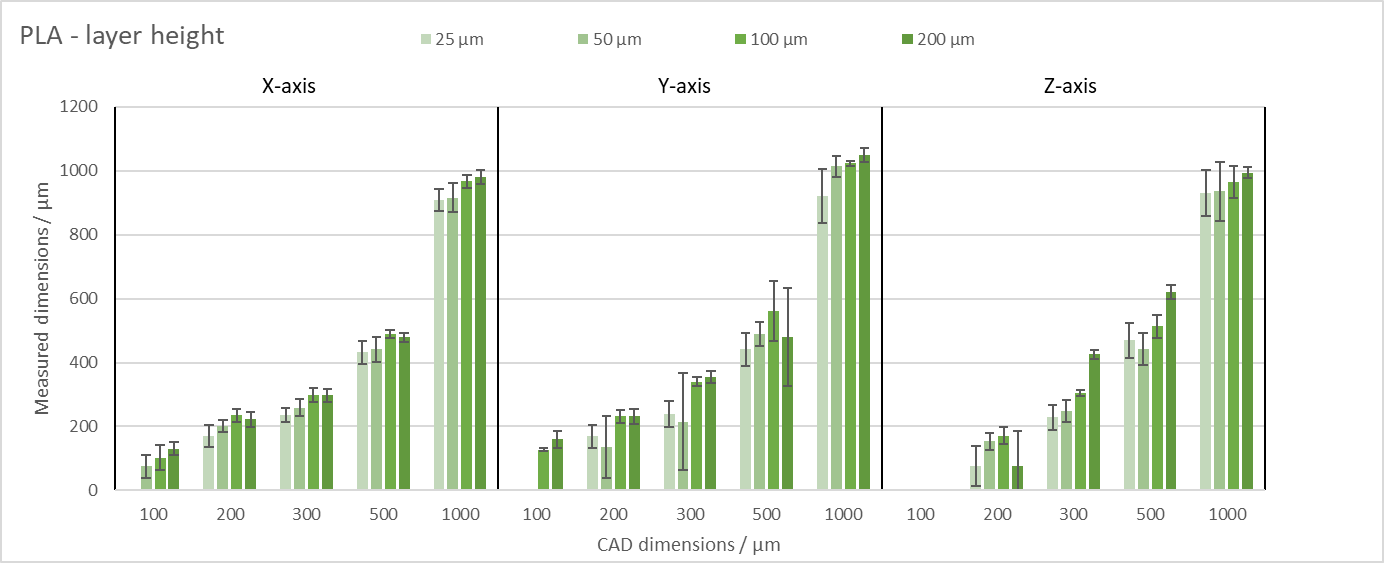


**Fig. S7| Obtained dimensions of the 3D printed poly (lactic acid) (PLA) test device for various layer heights.** Remaining parameters were kept constant at ϑ = 190 °C, v =70mm/sand fan = 100%. Results are shown for the X-, Y- and Z-axis separately. Values shown as mean ± standard deviation of 3 devices.


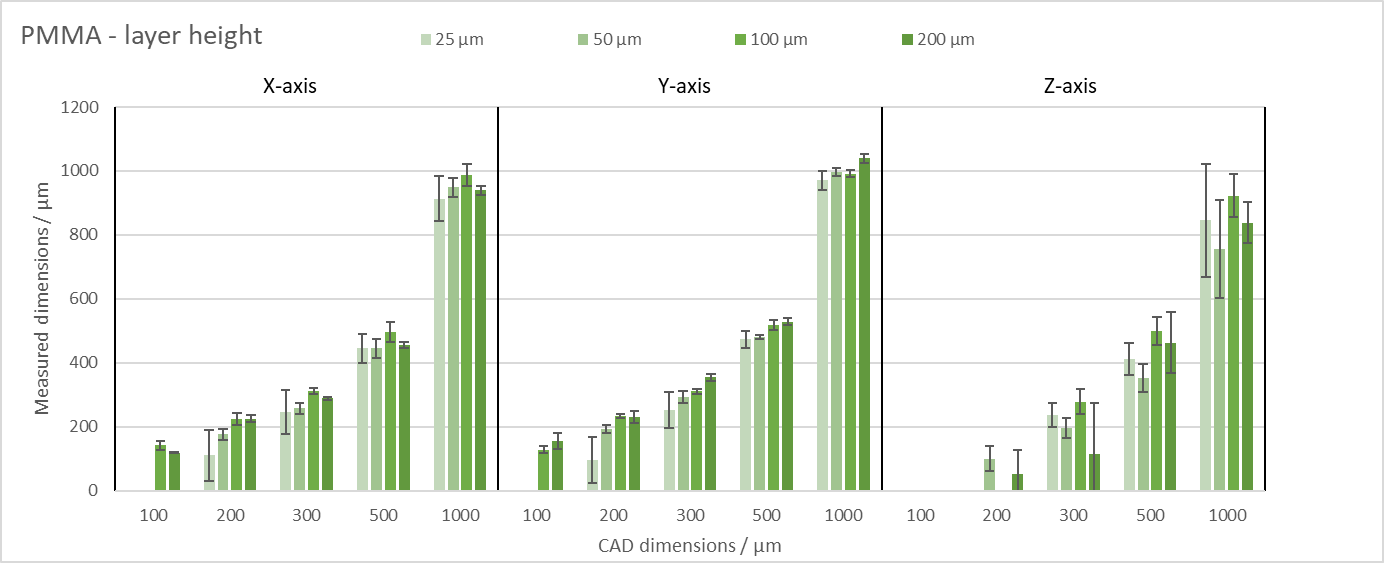


**Fig. S*8* | Obtained dimensions of the 3D printed poly (methyl methacrylate) (PMMA) test device for various layer heights.** Remaining parameters were kept constant at ϑ = 245 °C, v = 70 mm/s and fan = 50%. Results are shown for the X-, Y- and Z-axis separately. Values shown as mean ± standard deviation of 3 devices.


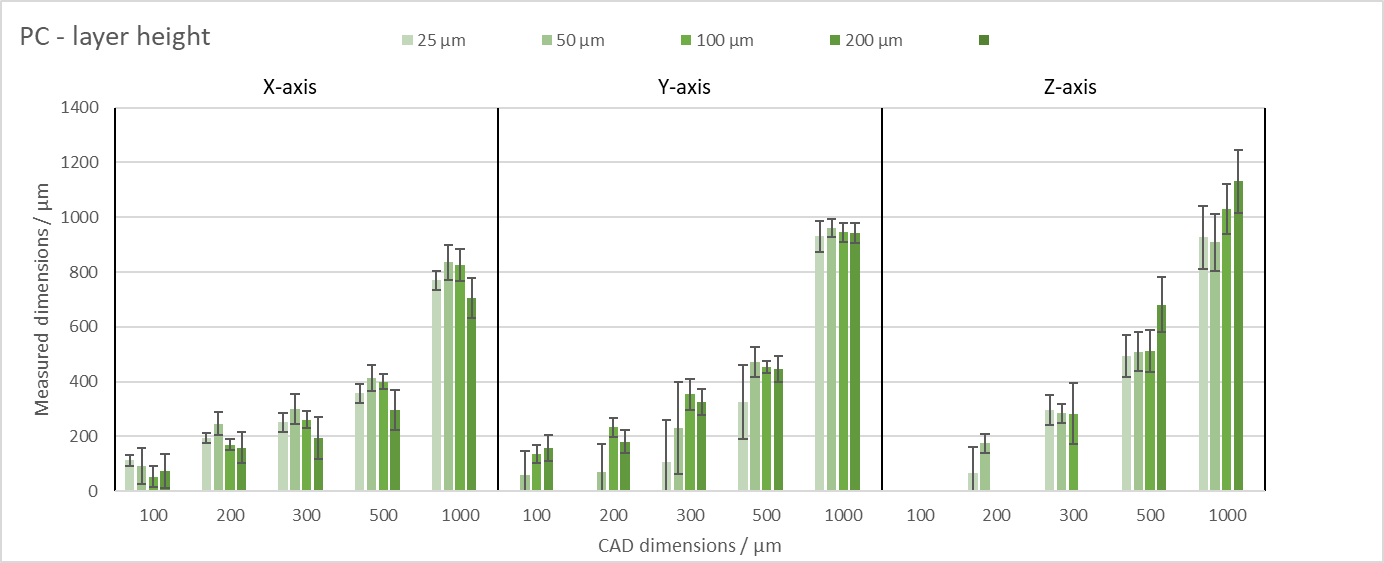


**Fig. S*9* |Obtained dimensions of the 3D printed polycarbonate (PC) test device for various layer heights.** Remaining parameters were kept constant at ϑ = 240 °C, v = 80 mm/s and fan = 0%. Results are shown for the X-, Y- and Z-axis separately. Values shown as mean ± standard deviation of 3 devices.

Analyzed parameter: fan speed (fan)


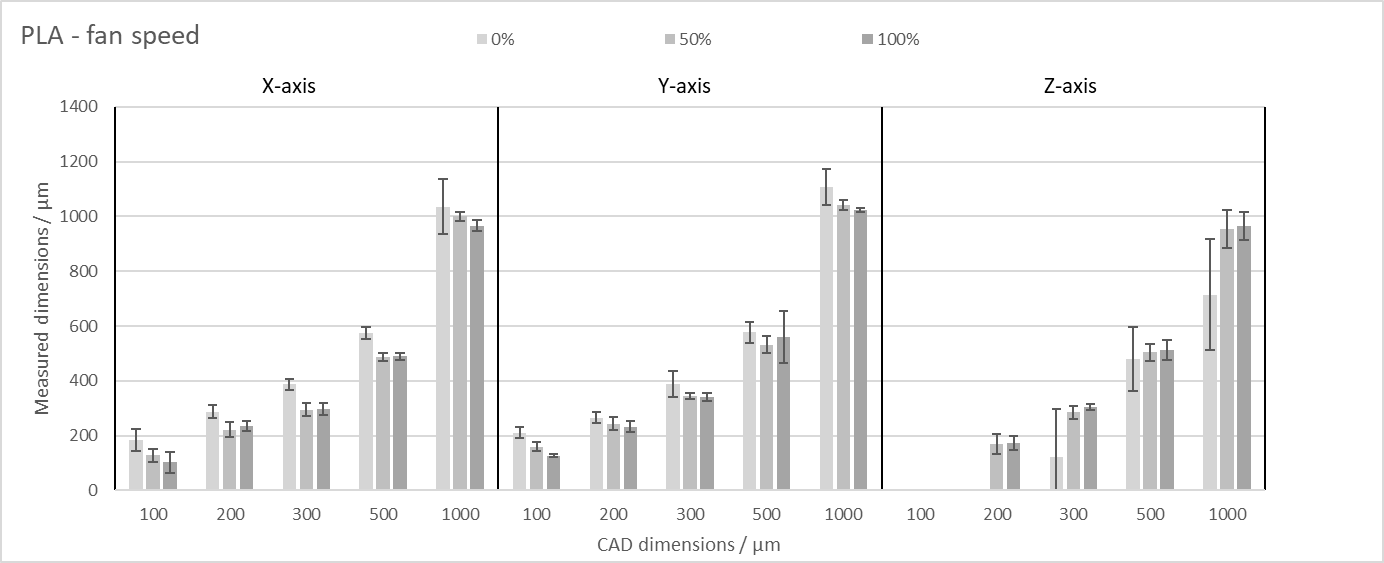


**Fig. S10| Obtained dimensions of the 3D printed poly (lactic acid) (PLA) test device for various fan speeds.** Remaining parameters were kept constant at ϑ = 190 °C, v = 70 mm/s and h = 100 µm. Results are shown for the X-, Y- and Z-axis separately. Values shown as mean ± standard deviation of 3 devices.


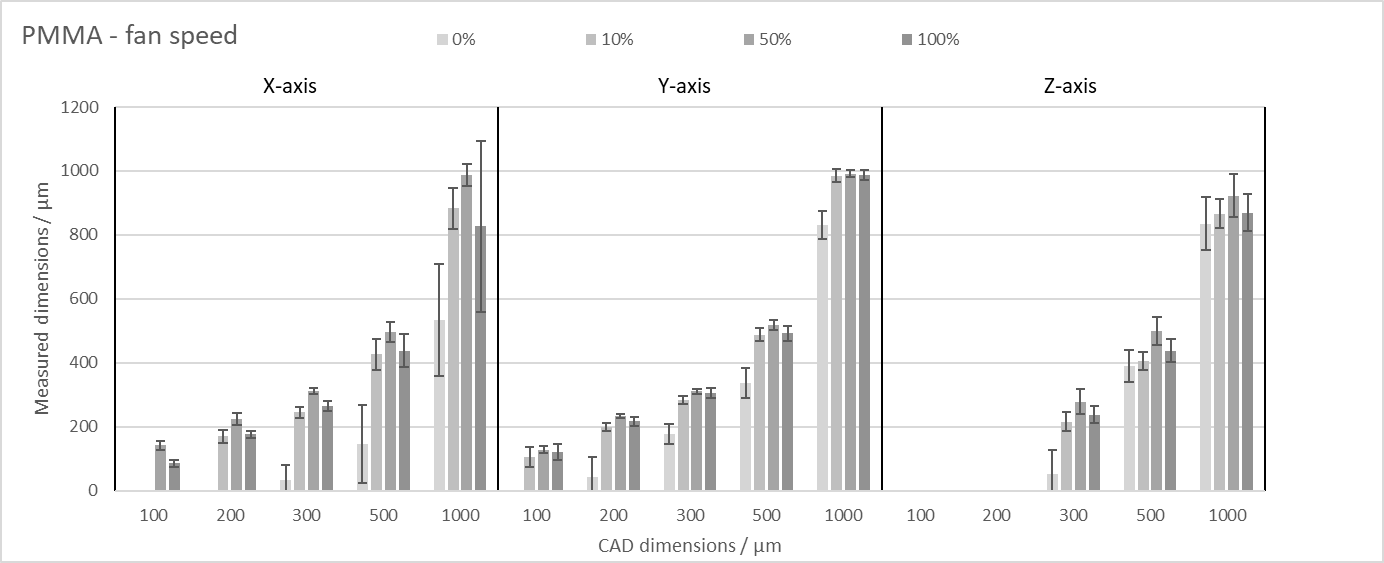


**Fig. S11|Obtained dimensions of the 3D printed poly (methyl methacrylate) (PMMA) test device for various fan speeds.** Remaining parameters were kept constant at ϑ = 245 °C, v = 70 mm/s and h = 100 µm. Results are shown for the X-, Y- and Z-axis separately Values shown as mean ± standard deviation of 3 devices.


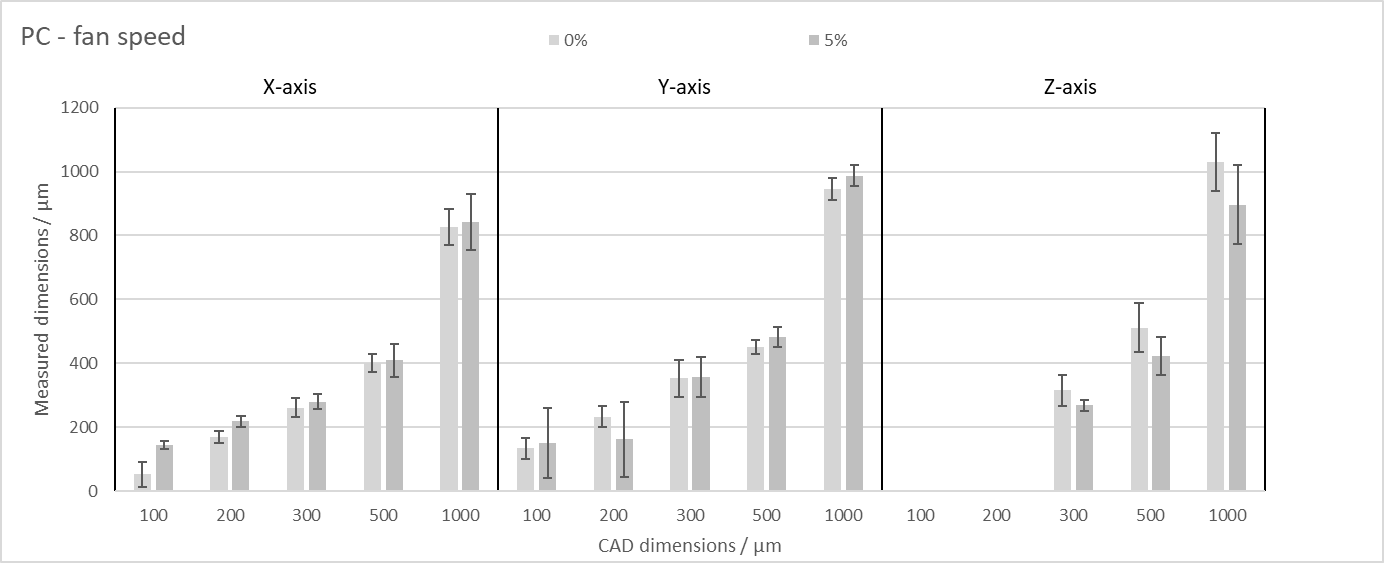


**Fig. S12| Obtained dimensions of the 3D printed polycarbonate (PC) test device for various fan speeds.** Remaining parameters were kept constant at ϑ = 240 °C, v = 80 mm/s and h = 100 µm. Results are shown for the X-, Y- and Z-axis separately. Values shown as mean ± standard deviation of 3 devices.

Microscopic images of the 3D-printed test devices

PLA test device


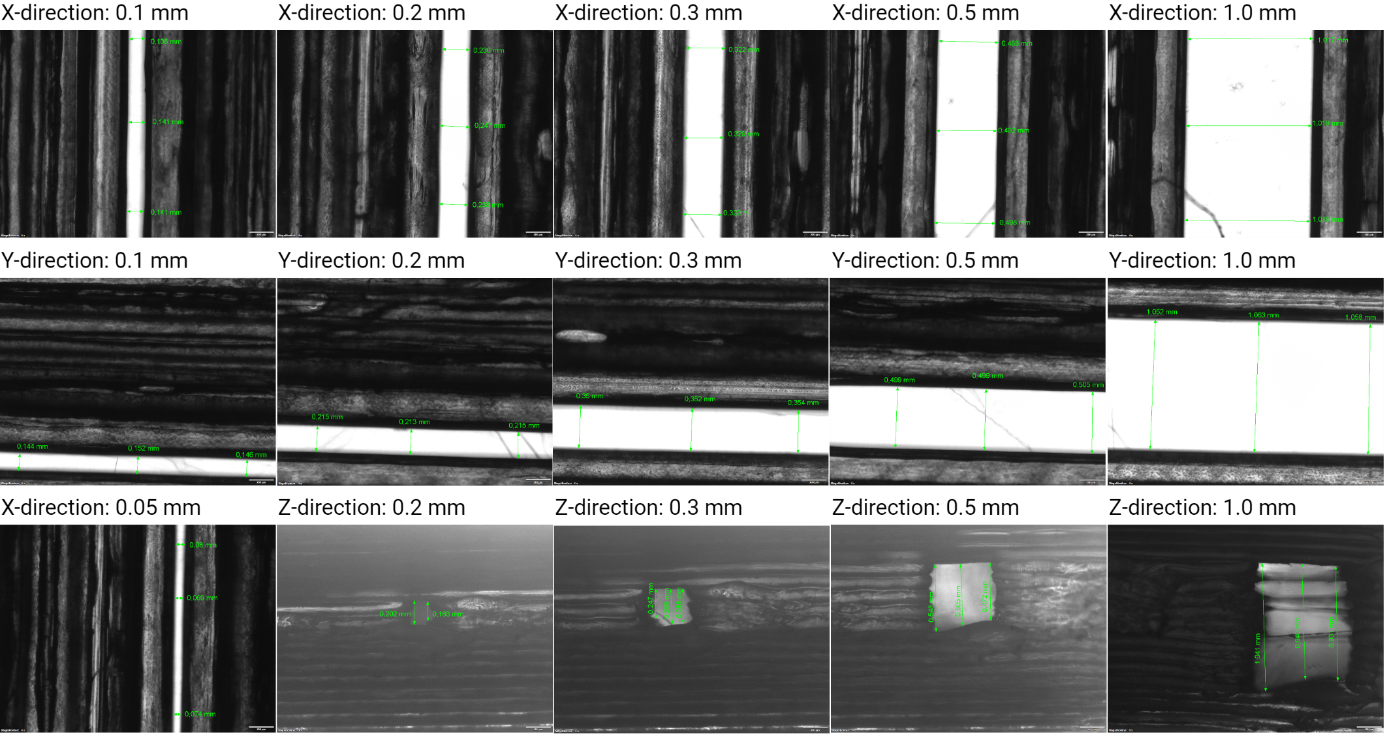


**Fig. S*13* | Representative microscopic images of FDM printed micro channels in a poly (lactic acid) (PLA) test device.** Analysis performed with “imageJ” (Version 1.52a, National Institutes of Health, USA). Scale bar measures 200 µm.

PMMA test device


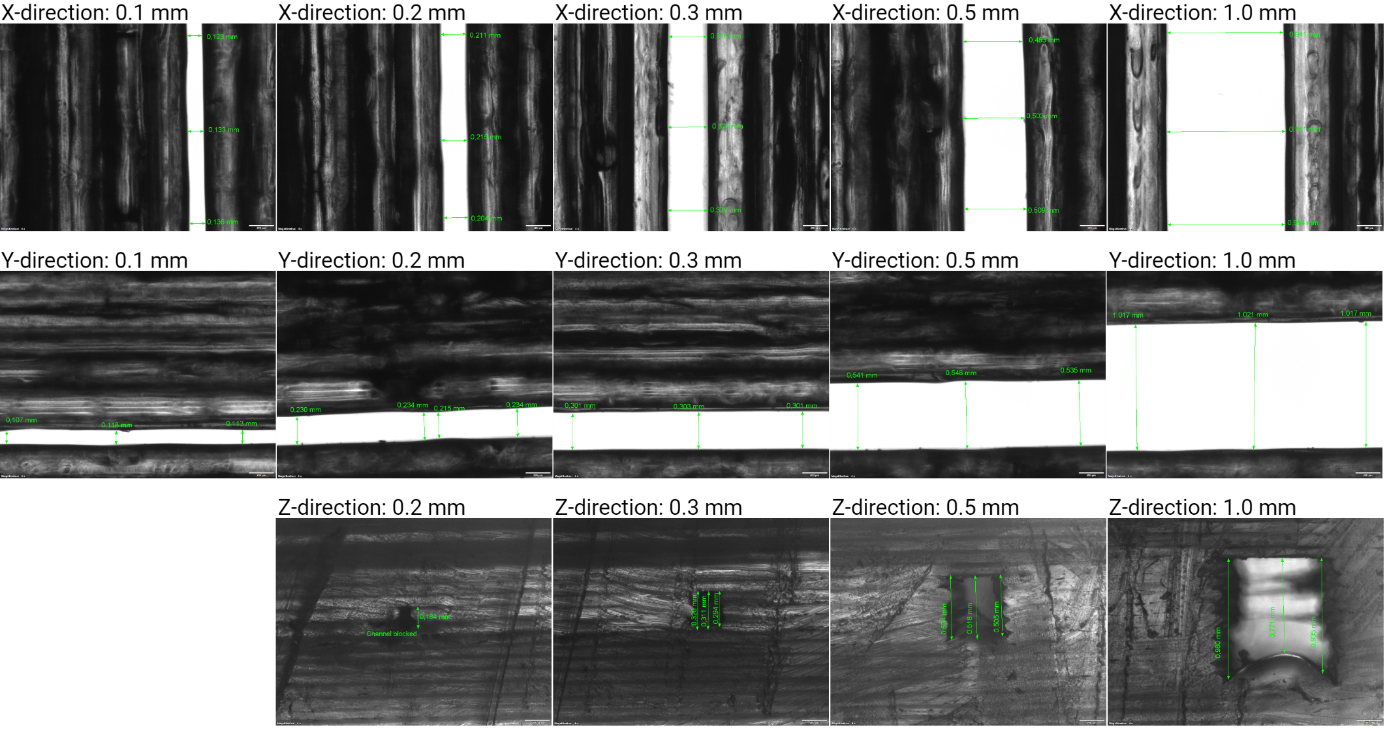


**Fig. S*14* |Representative microscopic images of FDM printed micro channels in a poly (methyl methacrylate) (PMMA) test device.** Analysis performed with “imageJ” (Version 1.52a, National Institutes of Health, USA). Scale bar measures 200 µm.

PC test device


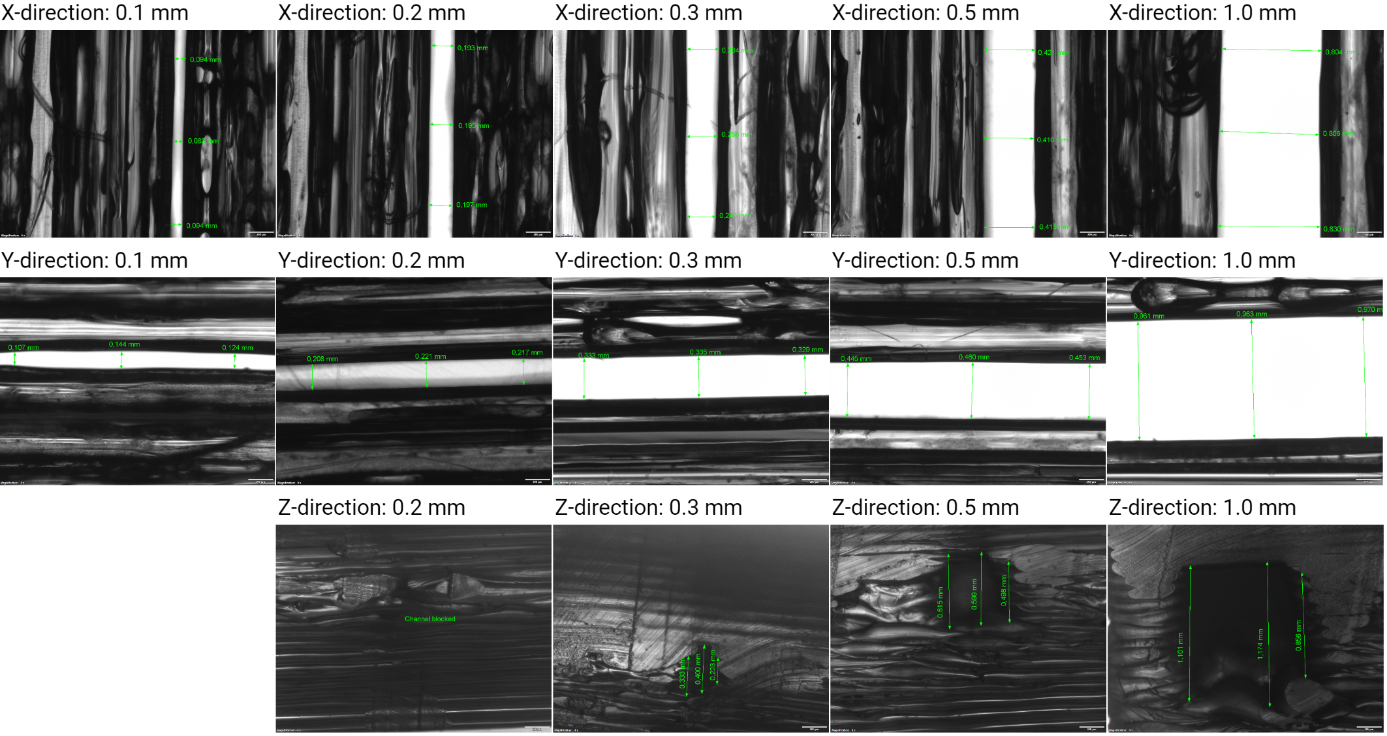


**Fig. S15|Representative microscopic images of FDM printed micro channels in a PC test device.** Analysis performed with “imageJ” (Version 1.52a, National Institutes of Health, USA). Scale bar measures 200 µm.

Relative deviation between experimental dimensions and CAD dimensions


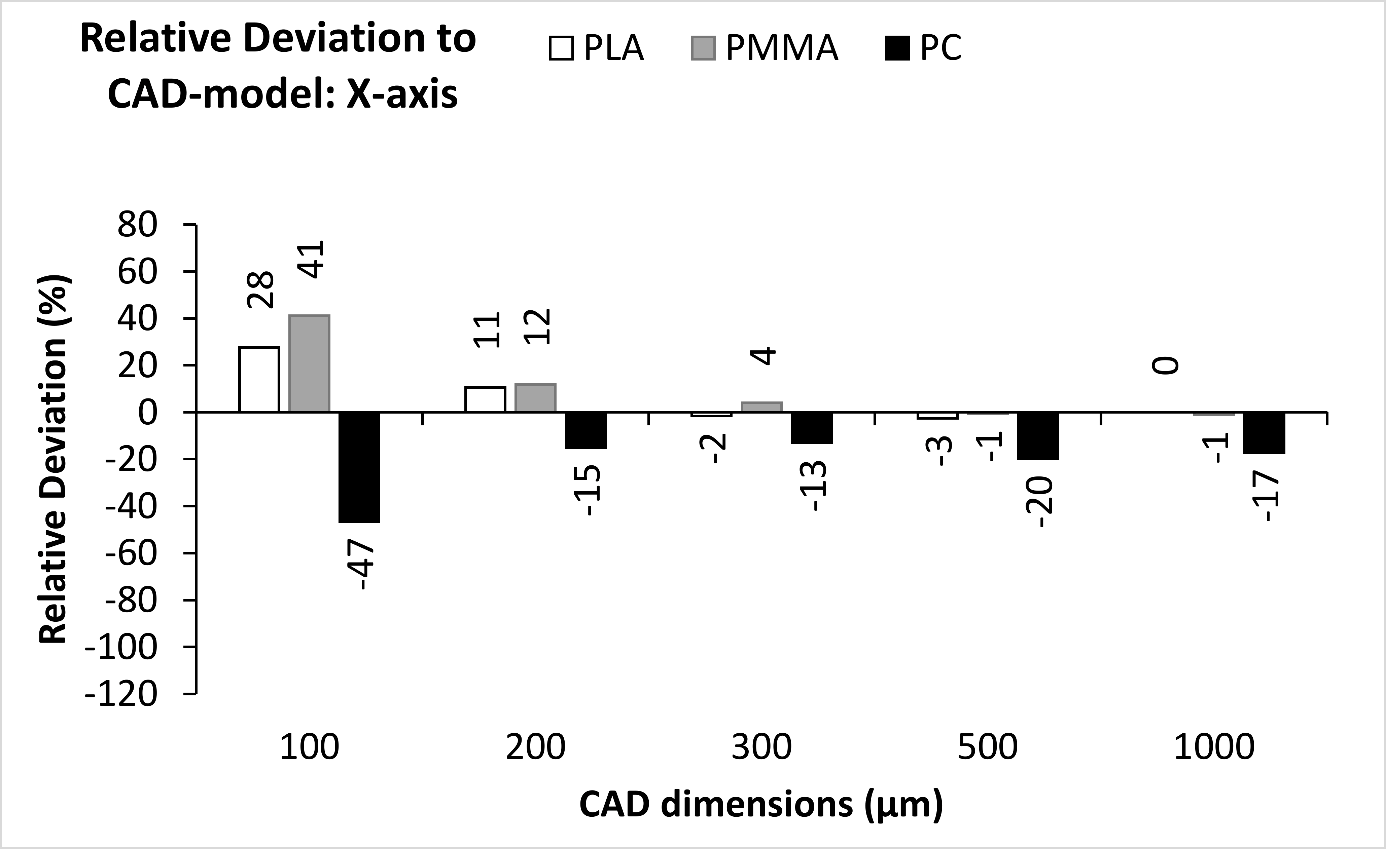


Fig.S16|Relative deviation between experimental dimensions and CAD dimensions in X-direction.


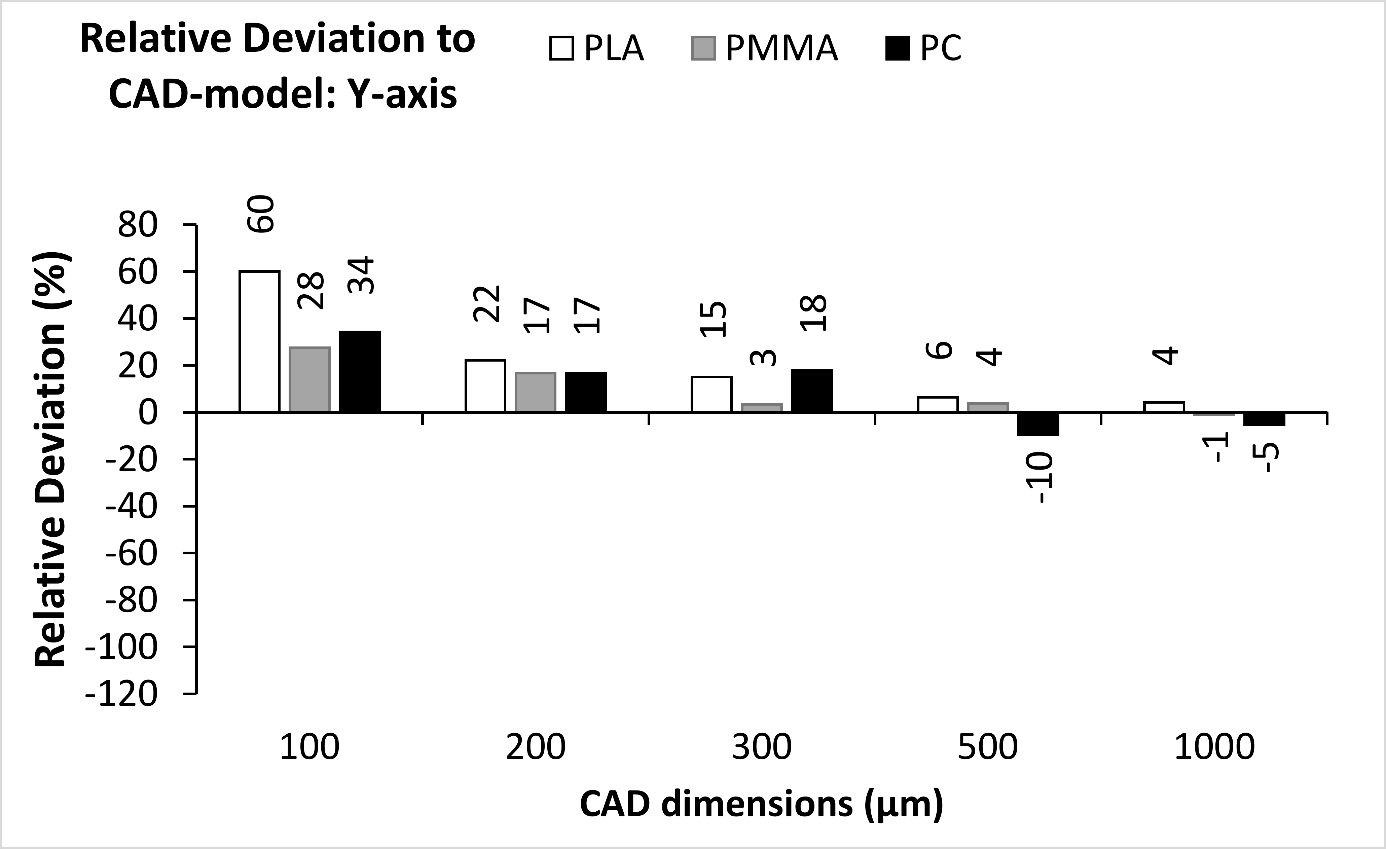


Fig.S17|Relative deviation between experimental dimensions and CAD dimensions in X-direction.


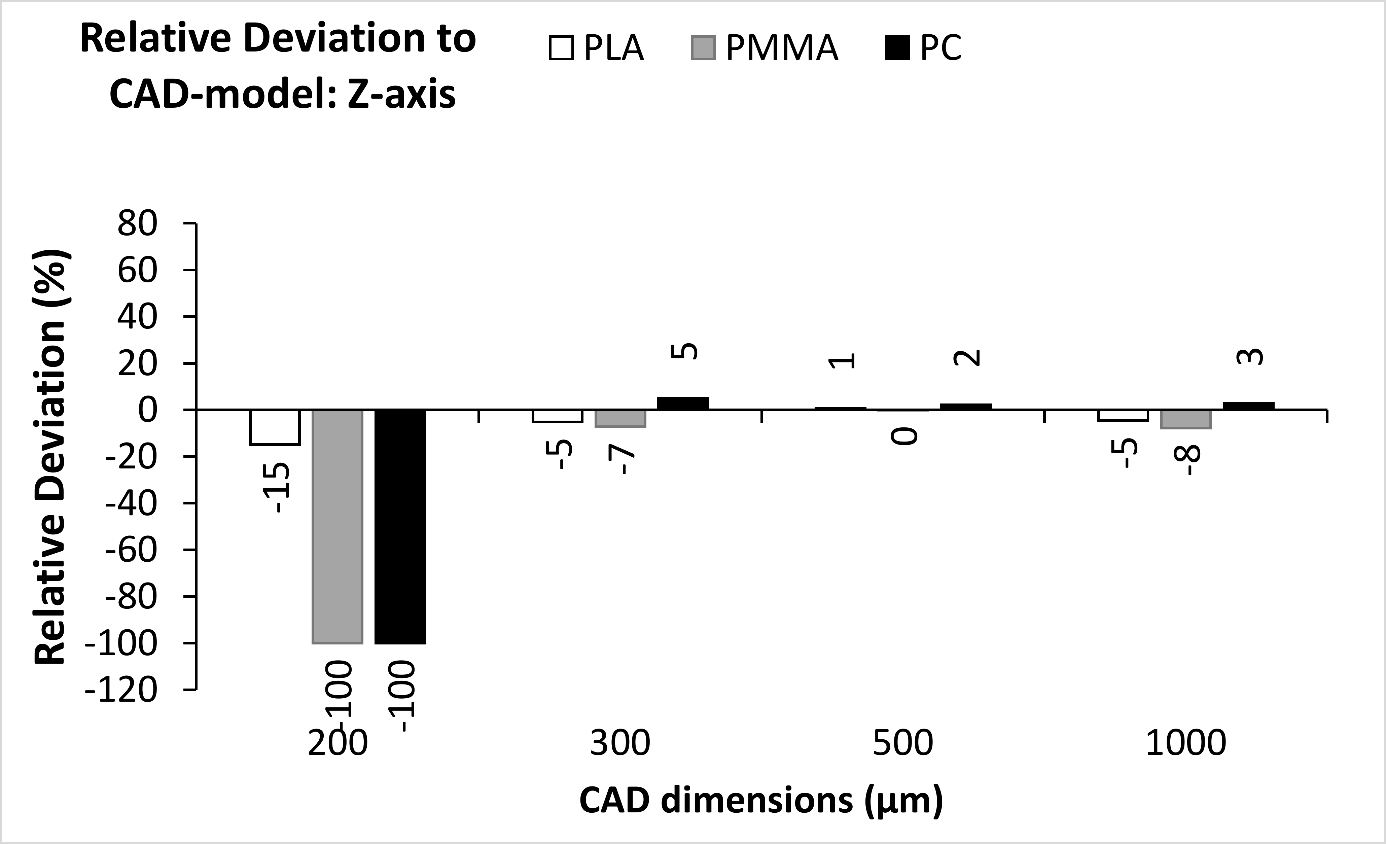


Fig.S18|Relative deviation between experimental dimensions and CAD dimensions in X-direction.

Biocompatibility


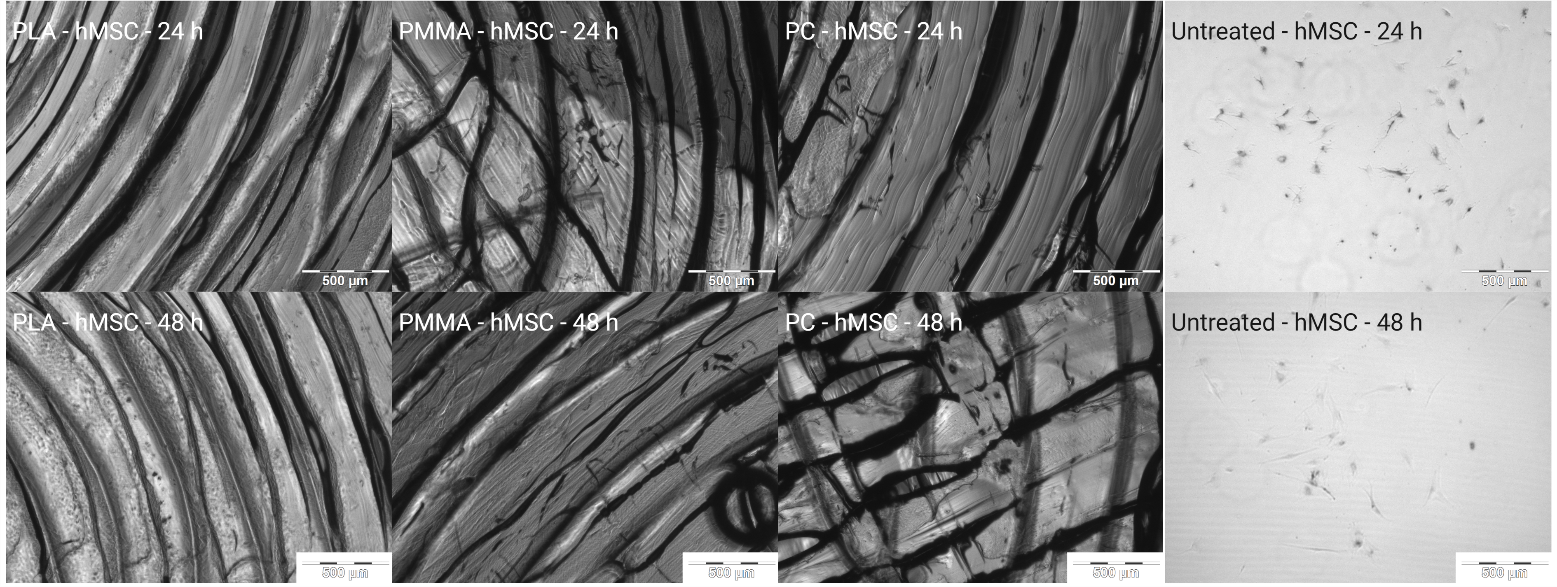


**Fig. S19|Representative microscopic images of FDM printed polymer discs cultivated with human mesenchymal stem cells (hMSC) for 24 and 48 hours.**Viability of hMSC cultivated on PLA, PMMA and PC discs was analyzed with MTT assay and compared to untreated hMSCs cultivated in a standard tissue culture 24-well plate. Images were taken after staining with MTT. Scale bar measures 500 µm.
